# Risk of mortality and cardiopulmonary arrest in critical patients presenting to the emergency department using machine learning and natural language processing

**DOI:** 10.1371/journal.pone.0230876

**Published:** 2020-04-02

**Authors:** Marta Fernandes, Rúben Mendes, Susana M. Vieira, Francisca Leite, Carlos Palos, Alistair Johnson, Stan Finkelstein, Steven Horng, Leo Anthony Celi

**Affiliations:** 1 IDMEC, Instituto Superior Técnico, Universidade de Lisboa, Lisbon, Portugal; 2 Hospital da Luz Learning Health, Lisbon, Portugal; 3 Hospital Beatriz Ângelo, Luz Saúde, Lisbon, Portugal; 4 MIT Critical Data, Laboratory for Computational Physiology, Harvard-MIT Health Sciences & Technology, Massachusetts Institute of Technology, Cambridge, Massachusetts, United States of America; 5 Institute for Data, Systems and Society, Massachusetts Institute of Technology, Cambridge, Massachusetts, United States of America; 6 Department of Emergency Medicine / Division of Clinical Informatics / Center for Healthcare Delivery Science, Beth Israel Deaconess Medical Center, Boston, Massachusetts, United States of America; 7 Division of Pulmonary Critical Care and Sleep Medicine, Beth Israel Deaconess Medical Center, Boston, Massachusetts, United States of America; Liverpool John Moores University, UNITED KINGDOM

## Abstract

Emergency department triage is the first point in time when a patient’s acuity level is determined. The time to assign a priority at triage is short and it is vital to accurately stratify patients at this stage, since under-triage can lead to increased morbidity, mortality and costs. Our aim was to present a model that can assist healthcare professionals in triage decision making, namely in the stratification of patients through the risk prediction of a composite critical outcome—mortality and cardiopulmonary arrest. Our study cohort consisted of 235826 adult patients triaged at a Portuguese Emergency Department from 2012 to 2016. Patients were assigned to emergent, very urgent or urgent priorities of the Manchester Triage System (MTS). Demographics, clinical variables routinely collected at triage and the patients’ chief complaint were used. Logistic regression, random forests and extreme gradient boosting were developed using all available variables. The term frequency–inverse document frequency (TF-IDF) natural language processing weighting factor was applied to vectorize the chief complaint. Stratified random sampling was used to split the data into train (70%) and test (30%) data sets. Ten-fold cross validation was performed in train to optimize model hyper-parameters. The performance obtained with the best model was compared against the reference model—a regularized logistic regression trained using only triage priorities. Extreme gradient boosting exhibited good calibration properties and yielded areas under the receiver operating characteristic and precision-recall curves of 0.96 (95% CI 0.95-0.97) and 0.31 (95% CI 0.26-0.36), respectively. The predictors ranked with higher importance by this model were the Glasgow coma score, the patients’ age, pulse oximetry and arrival mode. Compared to the reference, the extreme gradient boosting model using clinical variables and the chief complaint presented higher recall for patients assigned MTS-3 and can identify those who are at risk of the composite outcome.

## 1 Introduction

### 1.1 Emergency department triage

Emergency Department (ED) triage is the first evaluation of a patient when the patient condition and acuity level are defined. The main purpose of triage is to identify patients who need immediate care. ED triage systems ensure that patients are assigned a certain level of priority that is appropriate to their clinical condition and urgency of treatment. Thus, an accurate triage decision support system to assist the health professional becomes of vital importance.

### 1.2 Existing clinical triage systems

So far, several triage systems have been implemented worldwide to assist health professionals classifying patients according to the severity of their medical condition. The Manchester Triage System (MTS) is a 5-level triage system widely used in Europe [1]. MTS priorities range from level 1 (emergent patients that should have immediate medical observation) to level 5 (non urgent patients that should wait a maximum time of 4 hours for medical observation). Although this triage system is well established, over-triage and under-triage still occur, which indicates room for improvement. According to studies [2–5], under-triage contributes to delays in time-sensitive interventions, morbidity, and mortality. Over-triage, may have indirect but equally harmful effects [6], resulting in diversion of limited time and resources from more urgent patients and inappropriate allocation to less severe patients. In a study [2], the clinical severity of under-triage in the MTS was assessed for a cohort of pediatric patients. It was found that 0.9% (119/13408) patients were under-triaged and that in 53% (63/119) of these patients, experts considered under-triage as clinically severe. It was concluded that although infrequent, under-triage could have serious clinical consequences. In another study [6], the frequency of under- and over-triaged patients was measured by nurses using the Emergency Severity Index (ESI). Initial ESI-determined triage priority was classified as inaccurate for 16426 of 96071 patients. It was found that under-triage was associated with significantly higher admission rates and critical outcomes, while over-triage was associated with a lower rate of both.

### 1.3 Prior work in machine learning for risk stratification

There is a richness of information contained in electronic health records (EHR) stored in large databases that can be explored using machine learning to provide insights to assist providers in making informed decisions based on objective criteria [7]. In the literature machine learning models have been developed to assist in the stratification of patients for prioritization, according to their acuity level at the triage [8–23], and according to their risk of mortality [24–37], cardiac arrest [32–34], Intensive Care Unit (ICU) admission [27–30], hospital admission [9, 27, 38–53], need for critical care [9], ED revisits [45, 54], abnormal medical condition [17], acute morbidity and infectious diseases [31] and expected number of resources [55].

A few studies used MTS priority as modeling predictor along with other variables collected at triage, namely for prediction of hospital admissions [39–41] and mortality [26, 53]. In [39], a logistic regression and an artificial neural network model both yielded an AUROC of 0.86 (95% confidence intervals (CI) 0.85-0.86) for prediction of hospital admission. The authors developed a nomogram using the logistic regression and an automated software decision support system with a web interface based on the artificial neural network model. In [40], a logistic regression model was developed yielding an AUROC of 0.88 (95% CI 0.87-0.88) and in [41], a logistic regression, a decision tree and a gradient boosted machine model presented an AUROC of 0.85, 0.82 and 0.86, respectively, for prediction of hospital admissions. In [26], a logistic regression was developed to predict mortality within 30 days from information of gender, major disease by priority, Charlson’s comorbidity index, ICU admission, blood transfusion, troponin elevation, door-to-team and team-to-ward time. The MTS priority was found to be highly predictive of subsequent 30-day mortality ranging from 4.8% (MTS-5) to 46.1% (MTS-1). The results for both independent predictors of death within 30 days were: door-to-team times OR of 1.13 (95% CI 1.07-1.18) and team-to-ward times OR of 1.07 (95% CI 1.02-1.13). In [53] a logistic regression model yielded an AUROC of 0.91 (95% CI 0.89-0.93) from information of MTS priority at presentation, age, gender, comorbidities, functional status at presentation, mode of arrival, time of ED visit, type of specialty, physiological parameters at different times, level of consciousness, need for supplement oxygen, need for ventilation assistance, use of vasoactive and inotropic agents and initial laboratory markers in the ED. Other studies were performed for mortality prediction, where MTS priority was not used as modeling predictor. In [25] an area under the receiver-operating characteristic curve (AUROC) of 0.92 (95% CI 0.92-0.93) was obtained using logistic regression. The mortality predictors of the final model were age, prolonged capillary refill time, blood pressure, mechanical ventilation, oxygen saturation, Glasgow coma score and the diagnostic category of the Acute Physiology and Chronic Health Evaluation II (APACHE II)—a classification system for severity of disease. In [24], the authors used logistic regression as well and the AUROC for the internal validation set was 0.85 (95% CI 0.84-0.86) and for the external validation set was 0.84 (95% CI 0.82-0.85). The predictors of the model were age, gender, year, mode of arrival, triage priority and nine triage complaint codes (cardiac arrest, syncope/collapse, other cardiac, sepsis, other neurological, stroke/transient ischaemic attack, other respiratory, malignancy and malaise). In [27], for the combined prediction of mortality and ICU admission, the authors used four machine learning models: Lasso regression, random forest, gradient boosted decision tree, and deep neural networks. As the reference model, the authors created a logistic regression model using the five-level ESI. The machine learning models outperformed the reference model, AUROC of 0.86 (95%CI 0.85-0.87) in the deep neural network vs 0.74 (95%CI 0.72-0.75) in the reference model, with less under-triaged patients in ESI triage levels 3 to 5 (urgent to non-urgent). Predictors included demographics, mode of arrival, triage vital signs, chief complaints and comorbidities.

A more comprehensive systematic review of machine learning applied for ED triage decision support can be found in [56].

### 1.4 Goals of this investigation

In this work, we employed machine learning and natural language processing to identify ED patients with high risk of mortality and cardiopulmonary arrest using data routinely collected at triage from a Portuguese public Hospital Beatriz Ângelo (HBA). The case study of Portugal is relevant, given that it represents high demand for emergency services, compared to other Organisation for Economic Co-operation and Development (OECD) countries [57]. We developed models for prediction of a composite outcome combining mortality and cardiopulmonary arrest, in the first 24 hours after triage. The models were developed for the cohort of emergent, very urgent and urgent patients, where early intervention is more critical, compared to the cohort of standard, less urgent and non-urgent patients. The performance obtained with these models was compared against the reference model trained using only the MTS priority. The impact in the models performance was assessed when including the chief complaint text feature. The calibration of the models was also assessed, since inadequate model calibration can limit the use of the models for clinical decision support [58–62]. The uncertainty and reliability of risk predictions were finally assessed for the best model.

## 2 Materials and methods

### 2.1 Study overview

Data acquired from the Emergency Department Information Systems (EDIS) of HBA ranged from 2011 to 2016, with a total of 599276 ED visits in the adult population (≥ 18 years old). This study was approved by HBA Ethics Committee that waived the requirement for informed consent. At HBA, re-triage is performed for reassessment of parameters or change of priority, activation of clinical pathway or introduction/correction of other information in the system. re-triages were excluded from the study given that not all patients are re-triaged. Referrals from HBA ED to other hospital ED were excluded as well, since there was no information regarding the patients’ outcome. Patients who died before ED admission were also excluded. Finally, the cohorts of non-urgent or standard priority patients were excluded, as well as patients assigned a white priority (in HBA this priority is assigned for patients with less urgency for care). Less urgent patients usually are admitted to the ED for minor problems, and although there were no patients in this cohort with a critical outcome, it is a rare event in this cohort. The exclusion of these patients also reduced class imbalance, allowing for the development of more reliable models. For detailed exclusion criteria refer to S1 Appendix in supplementary materials.

### 2.2 Data collection and processing

We included as predictors variables routinely collected at triage (temperature, heart and respiratory rate, systolic, diastolic and mean arterial blood pressure, pulse oximetry SpO_2_ and pain scale), the chief complaint, glycemia levels, Glasgow coma scale (GCS), the priority assigned to the patient, the patient age and gender, mode of arrival (ambulance, walk-in), disabilities (stretcher, wheelchair or none), time of triage (weekday, hour and month), ED visit (first triage registered on the system or not), number and type of exams prescribed at triage (ophthalmology, otolaryngology, electrocardiogram, X-ray and orthopedic). For each physiological variable, two dummy indicator variables were created indicating abnormal/not abnormal (1/0) and missing/not missing (1/0) values. Detailed preprocessing can be depicted in S2 Appendix.

#### 2.2.1 Text pre-processing

Term frequency–inverse document frequency (TF-idf) was used for vectorization of the chief complaint. The number of words (unigrams/bigrams/trigrams) to select from each patient chief complaint as well as the total number of words to use from the training vocabulary are indicated in S2 Table. The chief complaint for HBA dataset consists of unstructured free text and it was subjected to lowercasing, a process of temporal normalization, tokenization, expansion of abbreviations and correction using Jaro-Winkler and stemming. Detailed preprocessing can be depicted in S2 Appendix.

### 2.3 Outcome measure

The primary outcome measure is defined as the composite of in-hospital death or cardiopulmonary arrest within 24 hours after triage, with triage as reference (0 min).

### 2.4 Model development

For model design and evaluation, we performed stratified random sampling, splitting 70% of data for train and 30% for test. The data preprocessing methodology is depicted in supplementary materials. We performed a stratified 10-fold cross validation (CV) in the training set using a randomized search for hyperparameter optimization. The models were trained and tested with the same dataset partitions, to allow for reproducibility. The configuration of the model with highest AUROC was selected as well as the corresponding threshold. This model was evaluated in the held-out test dataset. We performed 100 iterations of bootstrapping random sampling in 95% confidence intervals (CI) to measure variance in performance.

We compared results from logistic regression (LR), random forests regression bootstrap aggregation of decision trees (RFR) and extreme gradient boosting classifier (XGBoost). These algorithms were selected so we could compare a boosting classification technique—XGBoost, and a bagging regression technique—random forests with a more traditional technique—LR. LR is suitable for predicting a binary outcome variable, such as deceased/not deceased when there are no non-linear relationships. Random forests perform a randomized sampling process to train a set of individual decision trees where each aggregates the output to produce a single probabilistic prediction for each outcome. At the end, the majority vote is implemented for all of the tree outcomes [63]. XGBoost algorithm is an efficient supervised learning algorithm which is a variant of the original Gradient boosting method [64], having been very recently developed in [65]. XGBoost iteratively applies greedy search to find the optimal model structure by adding a split to the existing tree structure at each iteration. We performed a randomized search of hyperparameters using 10-fold cross validation in the training set. The hyperparameters search settings can be depicted in S2 Table.

Besides AUROC, we also assessed the area under the precision recall curve (AUPRC), the average precision (AP), which summarizes a precision-recall curve as the weighted mean of precisions achieved at each threshold, the sensitivity, also referred to as recall or the true positive rate (TPR), specificity or true negative rate (TNR) and precision or positive predictive value (PPV), which were used in previous studies [41, 47, 49]. We calculated the F1 score, which is suited for dealing with imbalanced datasets [66] and Cohen’s Kappa (*κ*) [67] to analyse interrater reliability [68]. Finally, we presented the standardized mortality ratio (SMR) which in this case represents the ratio between the observed number of composite outcomes (death or cardiopulmonary arrest) predicted by the model and the number of composite outcomes which would be expected.

We also evaluated the quality of the predictions using the Brier Skill Score (BSS). The BSS is based on the Brier Score (BS), a statistical index which can be used to validate predicted probabilities. BSS = 1 − BS/BSref, thus if the reference score (BSref) is evaluated, it results in a BSS of 0.0, which represents a no skill prediction. Negative values represent worse than no skill and values above 0.0 represent skillful predictions, with a perfect prediction value being 1.0 [69].

## 3 Results

### 3.1 Summary of patient population

**Fig 1:**
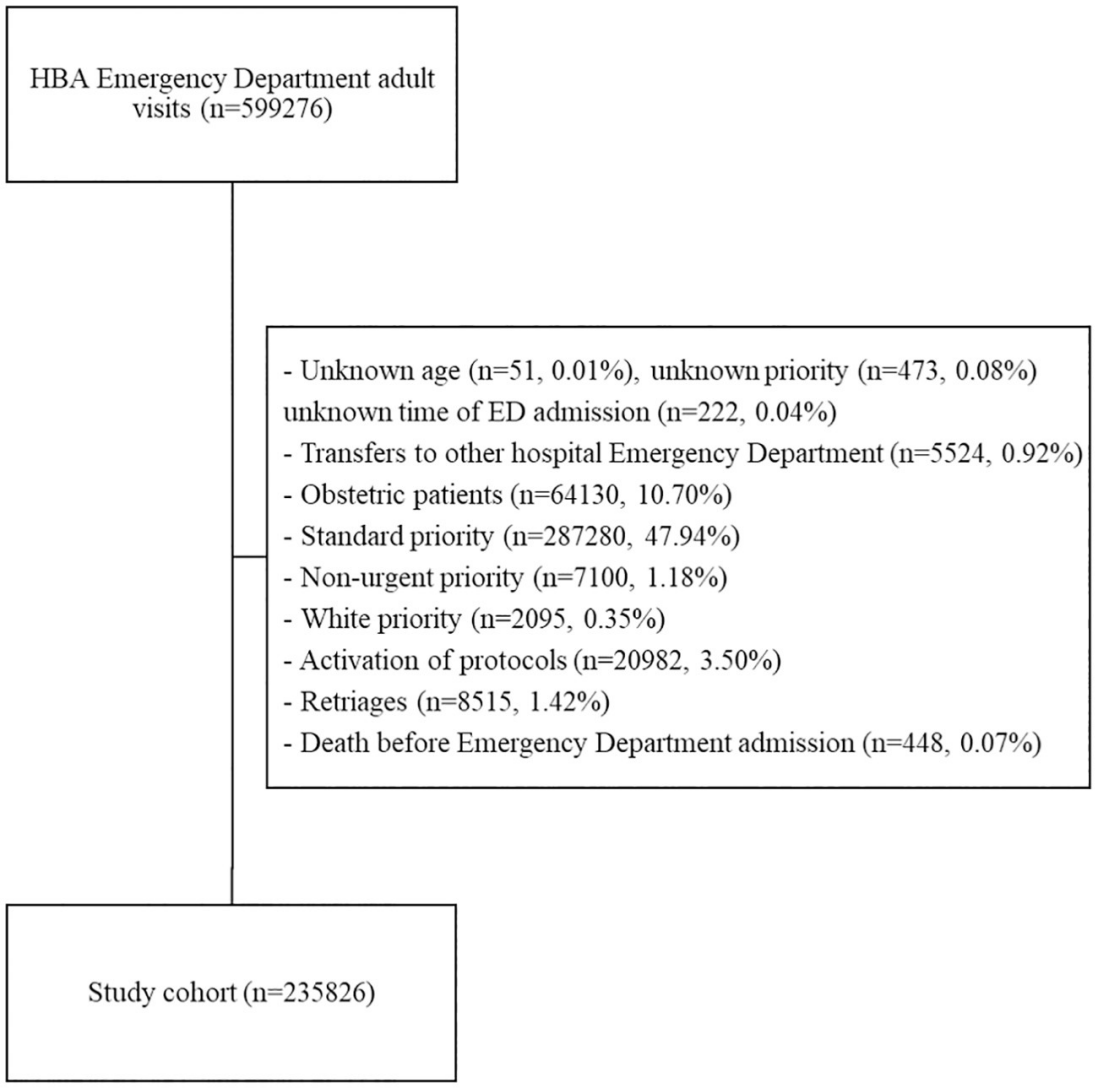
Inclusion and exclusion criteria. “n” corresponds to the number of triages.

Initially the dataset had a population of 599276 adult ED visits, from where we excluded triaged patients with unknown age (n = 51), unknown priority (n = 473) and unknown time of ED admission (n = 222). Standard (n = 287280), non-urgent (n = 7100) and white (n = 2095) priorities were excluded since the study focused on the first three most urgent priorities. Transfers to other hospital ED (n = 5524), obstetric patients (n = 64130), activation of protocols at triage (n = 20982), re-triages (n = 8515) and death before ED admission (n = 448) were also excluded leaving a final cohort of 235826 triaged patients, as presented in Fig 1. In this cohort, there were 1121 (0.48%) patients with a composite outcome: 1100 (98.13%) deceased, 21 (1.87%) with a cardiopulmonary arrest and 796 (71%) with both outcomes, in the first 24 hours after triage.

Demographics and a subset of variables are presented in Table 1. The descriptive statistics of the remaining variables used for modeling, namely number of missing and abnormal vitals, Glasgow coma scale and types of exams are presented in S3 Table, time variables in S4 Table, variables indicators of missing or abnormal values in vitals in S5 Table and pain scale statistics in S6 Table.

**Table 1:** Demographic variables and a subset of features are summarized for emergency department patients with and without the composite outcome.

	Composite outcome	
	Yes (N = 1121)	No (N = 234711)
Gender		
Female	610 (54)	130292 (56)
Male	511 (46)	104419 (44)
Age (years old)	80 (12.3), 83	58 (21.0), 59
Arrival mode		
Walk-in	102 (9)	124888 (53)
Ambulance	718 (64)	57016 (24)
Other	301 (27)	52807 (23)
Disability		
Stretcher	252 (22)	12500 (5.3)
Wheelchair	9 (1)	10325 (4.4)
None	860 (77)	211886 (90.3)
Heart rate (beats/min)	89 (23.5), 86	86(13.2), 86
Diastolic blood pressure (mmHg)	71 (14.1), 77	77 (8.5), 77
Systolic blood pressure (mmHg)	130 (25.6), 143	143 (16.4), 143
Pulse oximetry (%)	91 (7.7), 96	96 (2.4), 96
Glycaemia (mg/dL)	162 (68.4), 147	147 (42.4), 147
Temperature (°C)	36 (1.4), 37	37 (0.7), 37
Respiratory rate (breaths/min)	18 (4.8), 17	17 (1.7), 17
Number of exams		
0	1109 (98.9)	213073 (91)
1	10 (0.9)	14783 (6)
2	2 (0.2)	4651 (2)
3 or more	0 (0)	2204 (1)
Priorities		
Emergent (MTS-1)	385 (34)	978 (0.4)
Very urgent (MTS-2)	539 (48)	40677 (17.3)
Urgent (MTS-3)	197 (18)	193056 (82.3)
MTS discriminators (top 5)	Very low pulse oximetry—287 (26)	Moderate pain—82831 (35)
	Airway compromised—213 (19)	Pleuritic pain—14176 (6)
	Ineffective breathing—156 (14)	Sudden onset—12330 (5.3)
	Change of conscious status—102 (9)	Low pulse oximetry—11034 (4.7)
	Abnormal pulse—60 (5)	Severe pain—10867 (4.6)

The table shows number of patients. The figures in parentheses are the column percentages within each categorical variable for the respective outcome of admission. For continuous variables mean is presented with respective range in parentheses, followed by median: mean (range), median. MTS—Manchester Triage System.

We observed that gender was balanced and that the triaged population with a composite outcome—the positive class—was older compared with the negative class (80 vs 58 years old). The majority of the patients in the positive class arrived in ambulance (64%) while those in the negative class walked-in (53%). Most of the patients in both classes had no disability at arrival, however in the positive class 22% arrived in a stretcher vs. 5.3% in the negative class.

Approximately half (48%) of the patients in the positive class was assigned a very urgent priority (MTS-2), while the majority (82%) of patients in the negative class was assigned an urgent priority (MTS-3). The top 5 MTS discriminators assigned to the positive class were “very low pulse oximetry”, “abnormal heart rate”, “compromised airway” and “ineffective breathing” as well as “change in conscious status”. For the negative class, three discriminators were related with pain (“moderate”, “severe” and “pleuritic”), the other two were “low pulse oximetry” and “sudden onset”. Regarding vital signs, the main discrepancy between positive vs negative classes was blood pressure (systolic—130 vs 143, diastolic 71 vs 77), pulse oximetry (91 vs 96) and glycaemia (162 vs 147).

### 3.2 Main results

**Fig 2:**
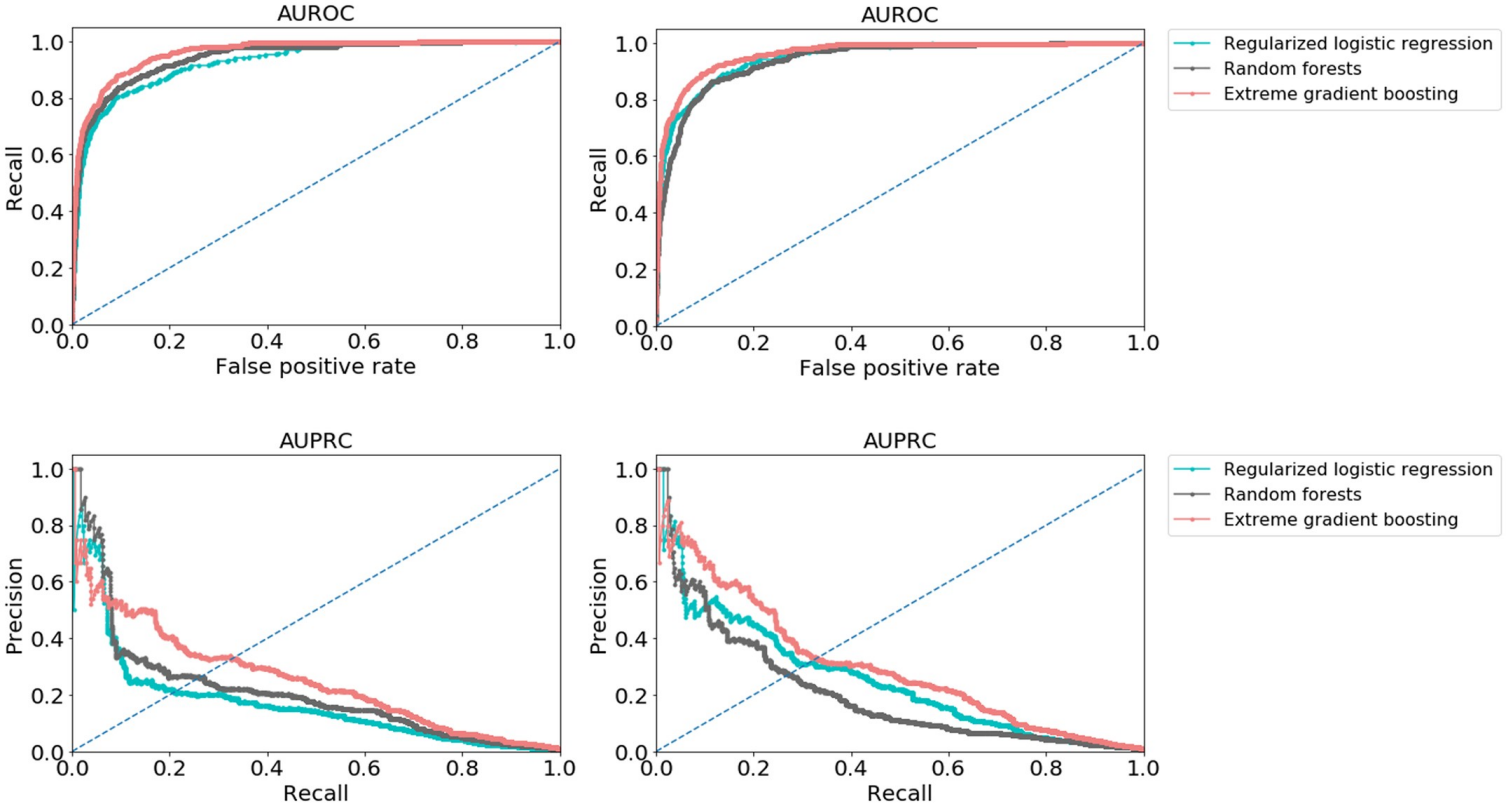
Models performance using only clinical variables (on the left) and including the chief complaint (on the right). For plots on the left, each model performance (AUROC, AUPRC) is: logistic regression (0.93, 0.17), random forests (0.95, 0.21) and extreme gradient boosting (0.96, 0.25). For plots on the right, each model performance (AUROC, AUPRC) is: logistic regression (0.95, 0.25), random forests (0.94, 0.20) and extreme gradient boosting (0.96, 0.30). AUROC—Area under the ROC curve. AUPRC—Area under the precision recall curve.

The modeling training set for negative and positive classes consisted of 164297 and 785 patients, respectively, and for the test set 70414 and 336 patients, respectively. We developed models using all available predictors except triage priority, since the priority assignment is subjective and can vary across providers. The difference in performance of the models including vs not including the chief complaint as predictor can be visualized in Fig 2. We observe that both AUROC and AUPRC yielded better results for LR and XGBoost models when this predictor was included. The increase in AUROC was 2% for LR and there was no change for XGBoost, however the AP increased in 8% and 4% for each model, respectively. Although RF presented better performance than LR when not using the chief complaint as predictor, when adding this predictor, LR presented higher performance. In terms of specificity and recall there was a 6% increase in recall and 3% decrease in specificity for LR, while for RF, on the contrary, recall decreased 1% and specificity increased 1%. For XGBoost, only the specificity increased 1%. Although LR presented a 0.86 recall vs 0.84 in XGBoost (a 2% difference), specificity for LR was 0.88 and for XGBoost 0.94 (a 6% difference). Despite this difference in sensitivity, we selected XGBoost as the best model since it presented higher overall performance compared to the other two models. The performance results for all models can be depicted in S7 Table.

**Fig 3:**
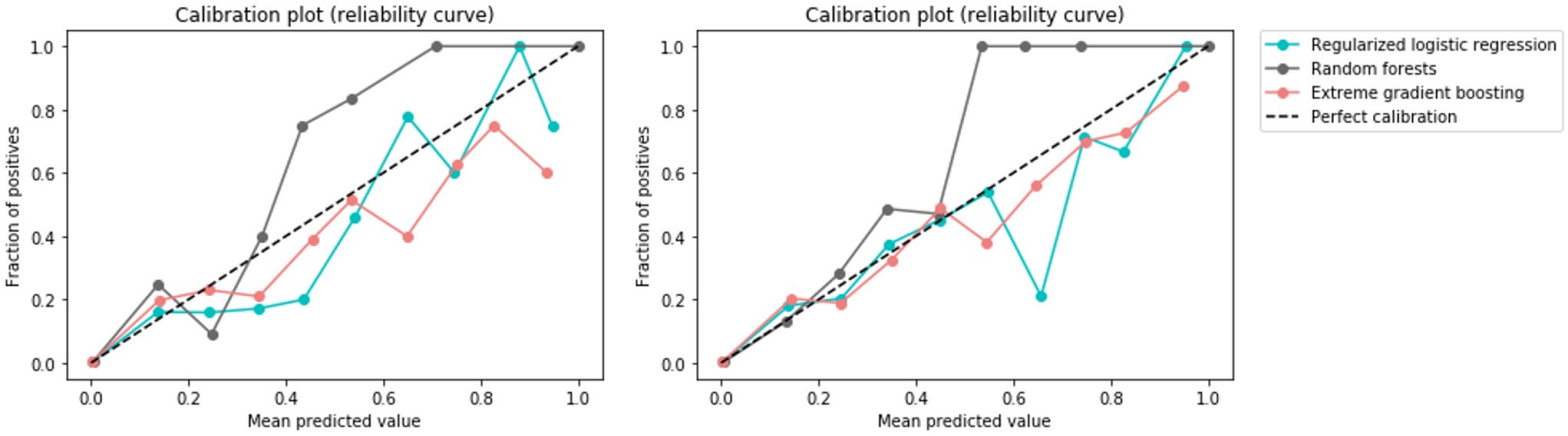
Calibration curves for the models using only clinical variables (on the left) and including the chief complaint (on the right).

**Fig 4:**
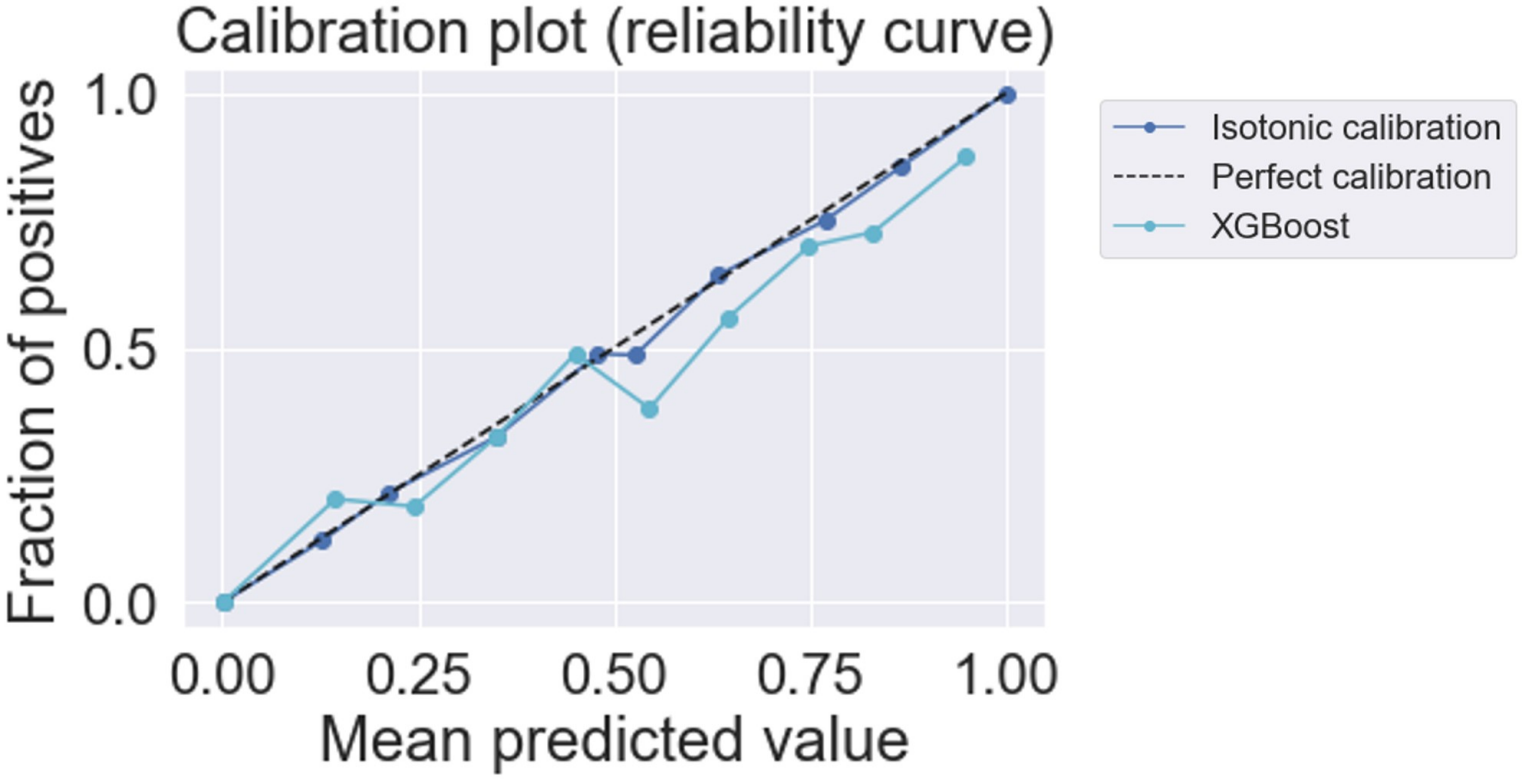
Calibration curve for the XGBoost model using clinical variables and the chief complaint with isotonic calibration.

We assessed the models calibration to understand the reliability of the predictions, as depicted in Fig 3. When the chief complaint was added, the calibration improved. RF did not exhibit a good calibration curve and under-predicted the risk probabilities while LR had the tendency to over-predict. The XGBoost model was better calibrated in patients on the lower end of the risk spectrum, but it became less well calibrated above the mean risk of 50% and as the risk increased. We applied isotonic calibration to the XGBoost model as depicted in Fig 4.

Among the models, the best was selected based on AUROC and AUPRC values and the calibration curves. XGBoost presented overall higher performance when compared to the other models. The results for this model with isotonic calibration can be depicted in Table 2, as well as the hyperparameters tuned in 10-fold CV during model training. The BSS, depicted in Table 3, also indicated a better quality of predictions given by XGBoost when compared to the other models. Analyzing the XGBoost hyperparameters, we observed that each tree was built upon the selection of 90% of the patients information and less than half (40%) of their features. A combination of unigrams and bigrams yielded the best performance using 1000 words from the training vocabulary. Comparing the XGBoost performance against the reference model using only triage priority as predictor, we observe a significant overall increase in all performance measures. There was an increase of 11% in average AUROC, 3% in AUPRC, 20% in AP, 12% in specificity and inter-rater agreement was also significantly higher. Sensitivity presented the lower increase (2%) among all measures. The threshold obtained for XGBoost was approximately a half of the one for the reference, which indicates the importance of finding the best threshold using a performance metric. Although the SMR was still high for the XGBoost model, when comparing to the reference, we observed a reduction to almost a third. We also compared the true and false predictions, given by the models against the real classifications for both XGBoost and the reference, which can be depicted in S3 Fig. Comparing to the reference, the XGBoost model presented lower number of false positive predictions and could better identify the risk of a composite outcome in urgent patients, correspondent to MTS-3.

**Table 2:** Performance results for the XGBoost calibrated model using clinical variables and chief complaint against the reference model (triage priority) and respective hyperparameters. AUROC was the performance measure for hyperparameter tuning and best model selection in train. The hyperparameters not mentioned in the table were the default in XGBClassifier from Python version 3.7.

Parameter	XGBoost	Reference
AUROC	0.96 [0.95-0.97]	0.85 [0.82-0.87]
AUPRC	0.31 [0.26-0.36]	0.28 [0.24-0.32]
AP	0.30 [0.25-0.34]	0.10 [0.07-0.12]
Specificity	0.94 [0.94-0.94]	0.82 [0.82-0.82]
Recall	0.84 [0.80-0.88]	0.82 [0.77-0.85]
Precision	0.06 [0.06-0.07]	0.02 [0.02-0.02]
F1	0.12 [0.11-0.13]	0.04 [0.04-0.05]
Cohen’s k	0.11 [0.10-0.13]	0.03 [0.03-0.04]
SMR	12.87 [11.69-14.20]	38.21 [34.77-42.29]
Threshold	0.008	0.015
Warm start	Yes	No
Regularization constant	*γ* = 5	C = 0.01
N-gram range	Combination of unigrams and bigrams	-
Words from vocabulary	1000	-
Number of trees	150	-
Maximum depth	4	-
Learning rate	0.09	-
Subsample (% of rows to build a tree)	90	-
Colsample (% of columns to build a tree)	40	-

In brackets is the result for 100 bootstrapping iterations in 95% confidence intervals. AUROC—area under the ROC curve. AUPRC—area under the precision recall curve. AP—Average precision. SMR—Standardized Mortality Ratio.

**Table 3:** Brier Skill Score (BSS) for the models using clinical variables and chief complaint against a unskilled reference model using triage priority. Reference Brier Score = 0.005.

	XGBoost	RF	LR	Reference
BSS	0.19	0.05	0.15	0.00

**Fig 5:**
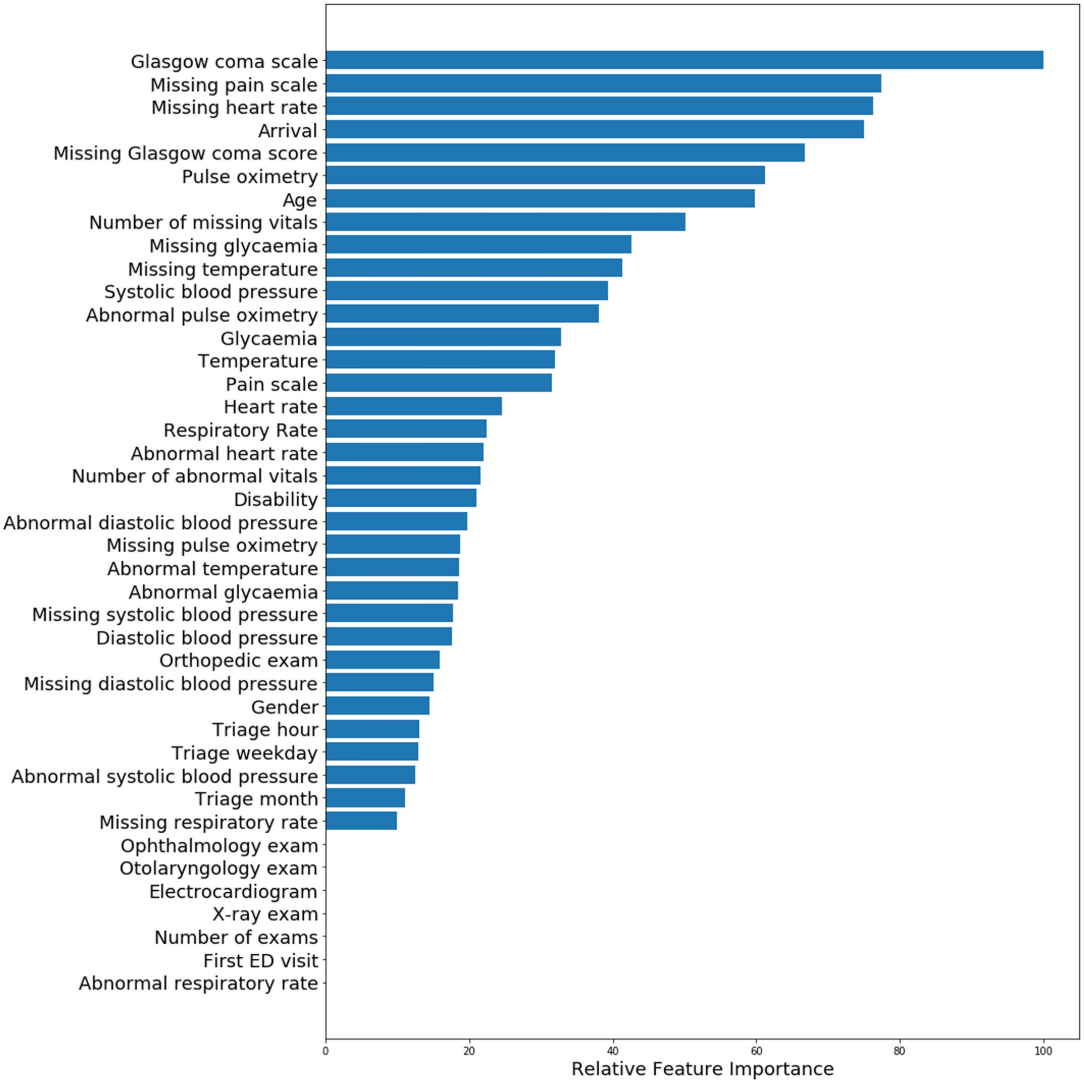
Relative importance of predictors obtained with XGBoost using all available variables. Exams are prescribed at the time of triage.

We assessed importance estimates of predictors in the train set given by each model. For LR, we used the absolute values of the regression coefficients, for RF and XGBoost we used the importance estimates given by the ensemble of trees. The XGBoost relative importance estimates is presented in Fig 5, and for LR and RF it can be depicted in S1 and S2 Figs. The most important predictor in the XGBoost was the Glasgow coma score. The patients’ arrival mode was ranked a relative importance of approximately 80% and the patients’ age and pulse oximetry were ranked an importance of 60%. The remaining predictors were ranked an importance inferior to 50%. From the information of exams ordered, only the orthopedic exam was ranked a relative importance of 18%, the remaining exams or number of exams were not considered important by the model. Regarding the time predictors, these were ranked with a lower importance (10-20%). For RF, the most important predictor was also the Glasgow coma score. Pulse oximetry was ranked an importance of approximately 55% and the remaining predictors were ranked an importance inferior to 50%. The most important predictor in the LR was the patients’ age. Pulse oximetry and systolic blood pressure were ranked an importance of approximately 50%, followed by temperature, number of exams and orthopedic exam. These were followed by diastolic blood pressure, which was also ranked as important by the XGBoost model. The models also ranked specific missing measured variables as important predictors of the composite outcome. Missing value of pain scale was ranked by XGBoost, LR and RF with an importance estimate of 80%, 70% and 20%, respectively. XGBoost model also ranked the missing values of heart rate and Glasgow coma score an importance of 80% and 70%, respectively.

**Fig 6:**
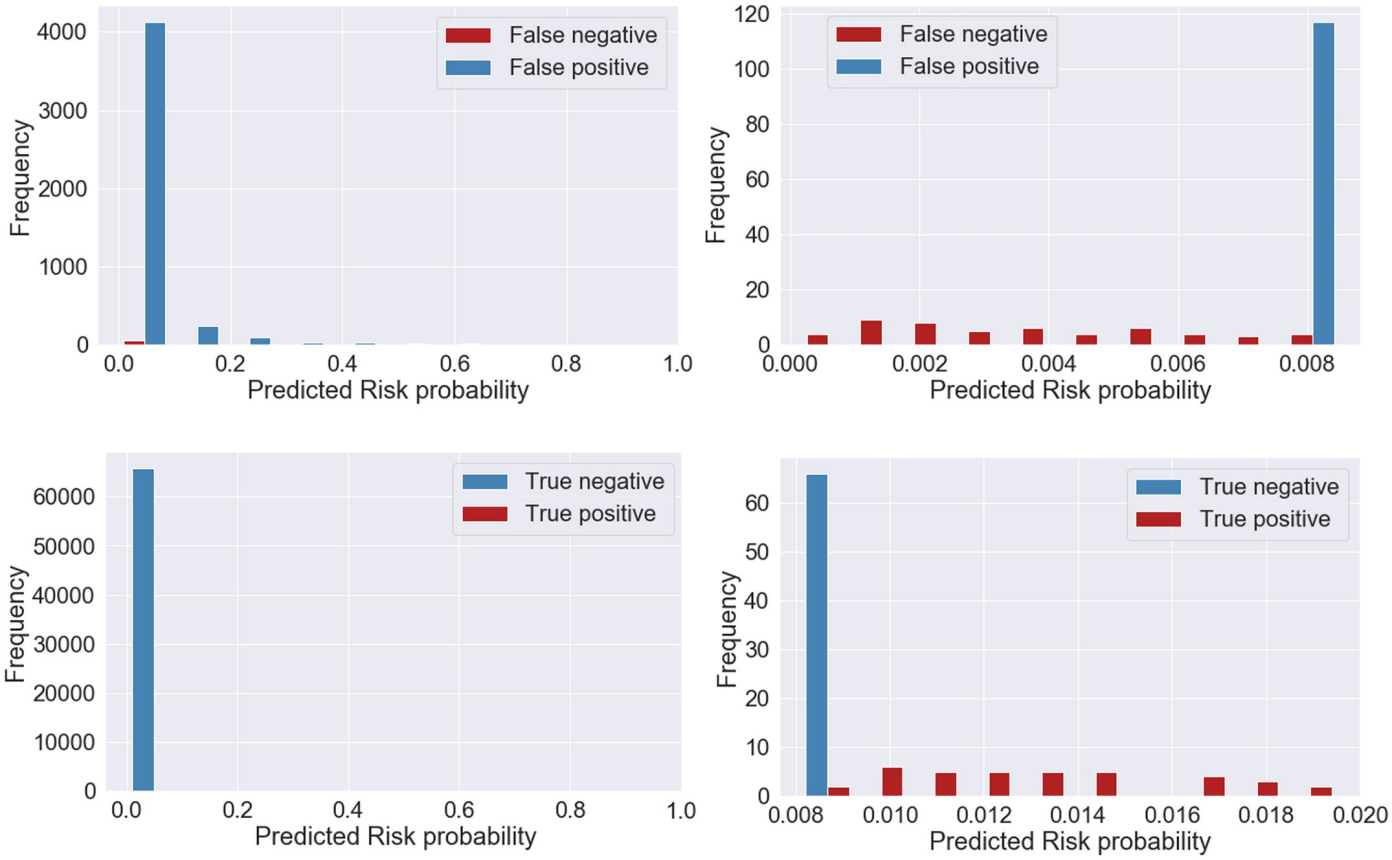
Risk probability for incorrect (on the top) and correct (on the bottom) classifications obtained with extreme gradient boosting model using clinical variables and chief complaint. A zoom-in is presented for the predicted risk probability for the plots on the right. The classification threshold is 0.00824.

**Fig 7:**
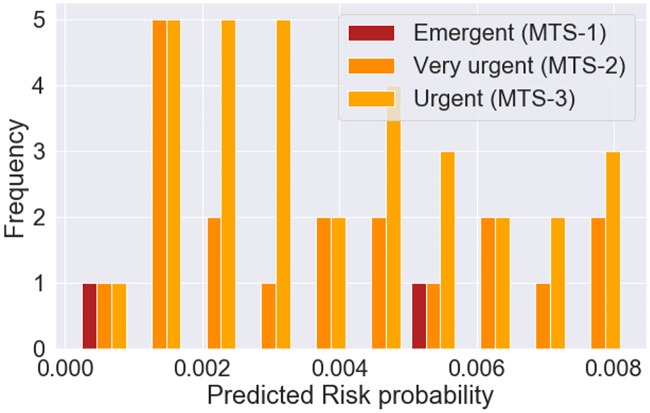
Predicted risk probability for false negative classifications discriminated by triage priority obtained with XGBoost model using clinical variables and chief complaint.

**Fig 8:**
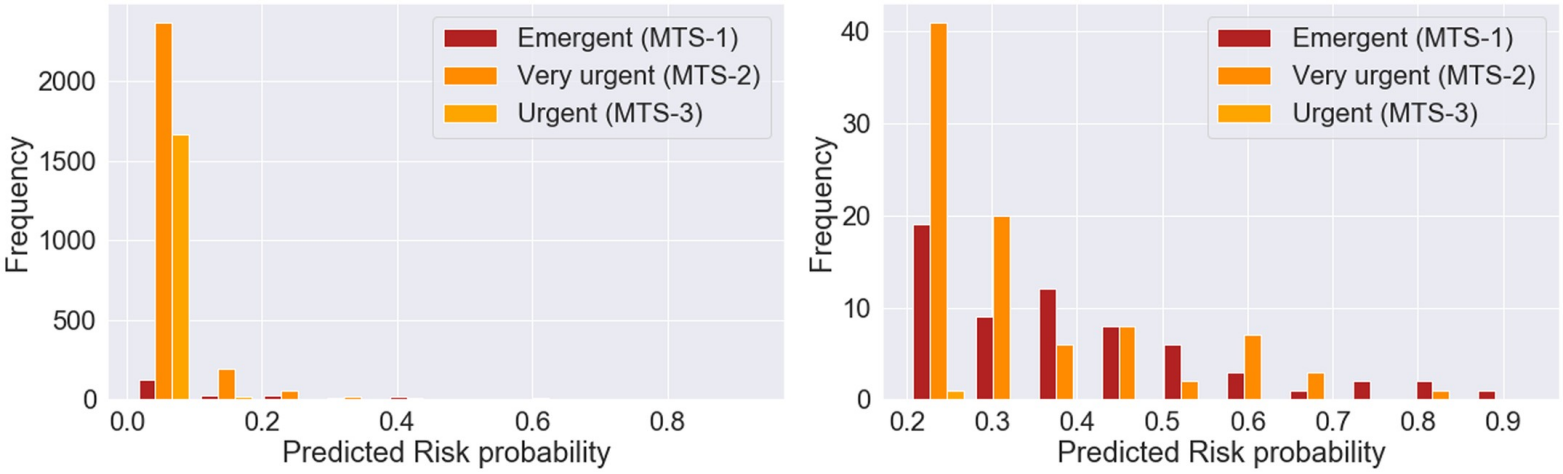
Predicted risk probability for false positive classifications discriminated by triage priority obtained with XGBoost model using clinical variables and chief complaint.

We finally evaluated the uncertainty in risk prediction given by the XGBoost model built with clinical variables and the chief complaint, through visualization of the predicted probability as presented in Fig 6. The threshold with the value of 0.00824 was selected using the training data, for the best model obtained in train based on the maximum value of AUROC. The higher the distance of the predicted probability to the threshold, the less uncertainty of the model prediction. For the false negative predictions (on top of Fig 6) this distance does not reach 1%. For the false positive predictions, the majority is located bellow 20% of distance to the threshold. For the true positives (on bottom of Fig 6) we observed that there was higher certainty in prediction, since the distances of the probabilities to the threshold were higher. For the true negative predictions, the maximum distance to threshold was bellow 1%. We also analyzed these results in a perspective of triage prioritization, for false negative and false positive classifications, as presented in Figs 7 and 8, respectively. For the case of false positive predictions, we observe that the highest distances to the threshold correspond to patients assigned an emergent priority (MTS-1 color red), while for the urgent priorities (MTS-3 color yellow) the uncertainty is higher since the probabilities are very close to the threshold and all bellow a distance of 20%. From this group of false positive patients, we found that 8% (n = 364) had a critical outcome in an interval of 48 hours after triage, either death, cardiopulmonary arrest, ICU or intermediate care unit admission, sepsis or stroke. In terms of uncertainty, for these 8% patients the absolute minimum, average and maximum distance to the threshold were 0.005%, 6% and 75%. Moreover, those with a distance superior to 20% corresponded to approximately 23% of the group of false positives. In terms of priorities, 14% corresponded to emergent patients with an average distance of 16%, 73% to very urgent patients with an average distance of 5% and 13% to urgent patients with an average distance of 2%.

## 4 Discussion

In this study we built a model to predict the risk of a composite critical outcome—mortality and cardiopulmonary arrest—in a cohort of emergent, very urgent and urgent patients who presented to the emergency department of a Portuguese hospital from 2011 to 2016. We observed that patients in the positive class were older and presented lower blood pressure, lower pulse oximetry and higher glycaemia levels. We developed models for prediction of the composite outcome in the first 24 hours after emergency department triage using regularized logistic regression, random forests regression and extreme gradient boosting. We assessed the models performance when adding the chief complaint—unstructured text feature—to the clinical and demographic variables. Including chief complaint as predictor increased the model predictive power for logistic regression and extreme gradient boosting. We concluded that although logistic regression presented 2% higher recall compared to extreme gradient boosting, the specificity was 6% higher for the latter model. Since we have a class imbalance this difference is significant, which is reflected in the other measures such as average precision, F1-score and Cohen’s *k*. When visualizing the models calibration curves, we observed that the calibration improved when adding the chief complaint. In both scenarios, random forests performed poorly, being under confident and focusing on the hard samples close to the classification threshold. An isotonic or sigmoid re-calibration could solve this issue, however it was not pursued since the other two models exhibited better calibration curves and overall performance. Logistic regression and extreme gradient boosting were better calibrated in patients on the lower end of the predicted risk spectrum, but became less well calibrated as the predicted risk increased, over-estimating the risk. We decided that it is preferable to over-estimate rather than under-estimate probabilities of a composite outcome for the high-risk group of patients. The extreme gradient boosting model presented the best calibration and overall performance results, therefore we proceeded the study using this model. We then compared its performance against the one obtained using only the priority assigned to the patients, with the Manchester Triage System. We concluded that the extreme gradient boosting model had a better predictive performance, exhibiting higher performance across all measures. Recall presented the lowest increase (2%), which indicates that the Manchester Triage System might lead to over-triage of patients. This is reflected in the higher value of standardized mortality ratio –- approximately three times higher than the gradient boosting model ratio. Compared to the reference, this model had the ability to identify patients with higher risk of a composite outcome in MTS-3 and presented lower number of false negative classifications for MTS-1 and MTS-2.

The important predictors of a composite outcome were identified for the models built using clinical variables and the chief complaint. We observed that for all models, the patients’ age, pulse oximetry and Glasgow coma score were ranked with relative high importance. The most important predictor for both extreme gradient boosting and random forests was Glasgow coma score. The most important predictor in the logistic regression was the patients’ age, which was also ranked with high importance by the other models. The arrival mode was also ranked a high importance by the extreme gradient boosting model. Since a significant amount of patients in critical conditions may arrive by ambulance, this can explain the ranking of importance given by the model to this predictor. The information regarding time for triage was not ranked a high importance by the this model, neither the number or type of exams, except for orthopedic exam which was ranked a relative low importance. Missing values of pain scale and Glasgow coma score were ranked a high importance. These variables might have been selected by the models as important predictors of the composite outcome due to the fact that the patients in severe conditions may be unresponsive, comatosed or unable to verbalize their symptoms and score their level of pain. The extreme gradient boosting model also ranked missing value of heart rate a relative high importance. The patient might have presented evident conditions of criticality or the intervention might have been immediate, and in these cases there was either no need or time to measure heart rate.

We finally assessed the uncertainty in risk prediction for the best model. A high uncertainty in the prediction of false positive classifications was observed, where the majority had a prediction very close to the threshold (bellow 5%). We observed that the predictions more far apart from the threshold corresponded to patients assigned an emergent priority. We hypothesize that these patients might have similar characteristics to those with a composite outcome, since they were assigned an emergent priority. However, the intervention might have been successful or on the other hand these patients might have had a composite outcome later than the 24 hours and therefore it was not accounted for our 24-hour outcome. We analyzed for these false positives if they had a composite outcome in the following 24 hours (48 hours after triage) and we concluded that 8% had an outcome of either death, cardiopulmonary arrest, ICU or intermediate care unit admission, sepsis or stroke. Furthermore, from this group of patients, the emergent presented the highest average distance to the threshold, which indicates less uncertainty in the prediction for this priority. When analyzing the correct model classifications, we observed that there was high certainty in the predictions of patients who had a composite outcome and for the majority of those who did not have the outcome, the frequency of predictions was higher for values far apart from the threshold, which corresponded to higher certainty.

We concluded that the measures of average precision and F1-score were relatively low compared to the other performance measures due to class imbalance present in the data. A significant proportion of patients did not have a composite outcome of death or cardiopulmonary arrest and may have had other complications, which the model does not take into account. To handle class imbalance we propose to use novelty detection methods as future work. Nonetheless, the extreme gradient boosting model presented good calibration and reliability in predictions. While the reference model presented higher recall for MTS-1 and MTS-2, the extreme gradient boosting model proved to have a higher sensitivity in identifying patients assigned MTS-3. This demonstrates the potential to complement the already existing triage system with a machine learning model and avoid under-triaging.

## Supporting information

S1 Appendix Detailed exclusion criteria. (PDF)

S2 Appendix Data pre-processing. (PDF)

S1 Table Criteria to exclude outliers and identify abnormal values in patients’ variables. DBP—diastolic blood pressure. (1) values not within normal range.
(PDF)

S2 Table Hyperparameter optimization in random search 10-fold cross validation. “Warm start” (reusing the solution of the previous call to fit as initialization) was varied between True/False for the three modeling techniques.
(PDF)

S3 Table Number of missing and abnormal vitals, Glasgow coma scale, first visit indicator and types of exams used for modeling summarized for emergency department patients with and without the composite outcome. (PDF)

S4 Table Time variables used for modeling summarized for emergency department patients with and without the composite outcome. (PDF)

S5 Table Abnormal and missing values for vital signs and pain scale used for modeling summarized for emergency department patients with and without the composite outcome. (PDF)

S6 Table Pain scale used for modeling summarized for emergency department patients with and without the composite outcome. (PDF)

S7 Table Average modeling performance results in test. In brackets is the result for 100 bootstrapping iterations in 95% confidence intervals.
(PDF)

S1 Fig Relative importance estimates obtained for logistic regression model using clinical variables and the chief complaint. (TIF)

S2 Fig Relative importance estimates obtained for random forests model using clinical variables and the chief complaint. (TIF)

S3 Fig Confusion matrices for the XGBoost model using clinical variables and chief complaint and for the reference model using triage priority. (TIF)

## References

[1] Mackway-Jones Kevin, Marsden Janet, and Windle Jill, eds. Emergency triage: Manchester triage group. John Wiley & Sons, 2014.

[2] Seiger N, van Veen M, Steyerberg EW, Ruige M, Van Meurs AH, Moll HA. Undertriage in the Manchester triage system: an assessment of severity and options for improvement. Archives of disease in childhood. 2011 7 1; 96(7):653–7. 10.1136/adc.2010.206797 21459879

[3] Hitchcock M, Gillespie B, Crilly J, Chaboyer W. Triage: an investigation of the process and potential vulnerabilities. Journal of advanced nursing. 2014 7; 70(7):1532–41. 10.1111/jan.12304 24372354

[4] Yurkova I, Wolf L. Under-triage as a significant factor affecting transfer time between the emergency department and the intensive care unit. Journal of Emergency Nursing. 2011 9 1; 37(5):491–6. 10.1016/j.jen.2011.01.016 21549418

[5] Haas B, Gomez D, Zagorski B, Stukel TA, Rubenfeld GD, Nathens AB. Survival of the fittest: the hidden cost of undertriage of major trauma. Journal of the American College of Surgeons. 2010 12 1; 211(6):804–11. 10.1016/j.jamcollsurg.2010.08.014 21036070

[6] Hinson JS, Martinez DA, Schmitz PS, Toerper M, Radu D, Scheulen J, et al Accuracy of emergency department triage using the emergency severity index and independent predictors of under-triage and over-triage in Brazil: a retrospective cohort analysis. International journal of emergency medicine. 2018 12 1; 11(1):3 10.1186/s12245-017-0161-8 29335793 PMC5768578

[7] Nair S, Hsu D, Celi LA. Challenges and opportunities in secondary analyses of electronic health record data In Secondary Analysis of Electronic Health Records 2016 (pp. 17–26). Springer, Cham. 31314274

[8] Azeez D, Ali MA, Gan KB, Saiboon I. Comparison of adaptive neuro-fuzzy inference system and artificial neutral networks model to categorize patients in the emergency department. SpringerPlus. 2013 12 1; 2(1):416 10.1186/2193-1801-2-416 24052927 PMC3776083

[9] Levin S, et al Machine-learning-based electronic triage more accurately differentiates patients with respect to clinical outcomes compared with the emergency severity index. Annals of emergency medicine. 2018 5 1;71(5):565 -74. 10.1016/j.annemergmed.2017.08.005 28888332

[10] Lin WT, Wu YC, Zheng JS, Chen MY. Analysis by data mining in the emergency medicine triage database at a Taiwanese regional hospital. Expert Systems with Applications. 2011 9 1; 38(9):11078–84. 10.1016/j.eswa.2011.02.152

[11] Zmiri D, Shahar Y, Taieb-Maimon M. Classification of patients by severity grades during triage in the emergency department using data mining methods. Journal of evaluation in clinical practice. 2012 4; 18(2):378–88. 10.1111/j.1365-2753.2010.01592.x 21166962

[12] Velarde ES, Sotelo-de Ávila AA, Rico-Asención IO, Alemán NR, González RS, Ramírez-Sotelo MG, et al Fuzzy-state machine for Triage priority classifier in emergency room In World Congress on Medical Physics and Biomedical Engineering, June 7-12, 2015, Toronto, Canada 2015 (pp. 1488–1491). Springer, Cham.

[13] Aziz D, Ali MM, Gan KB, Saiboon I. Initialization of adaptive neuro-fuzzy inference system using fuzzy clustering in predicting primary triage category. In 2012 4th International Conference on Intelligent and Advanced Systems (ICIAS2012) 2012 Jun 12 (Vol. 1, pp. 170-174). IEEE.

[14] Azeez D, Gan KB, Ali MA, Ismail MS. Secondary triage classification using an ensemble random forest technique. Technology and Health Care. 2015 1 1; 23(4):419–28. 10.3233/THC-150907 25791174

[15] Georgopoulos VC, Stylios CD. Fuzzy cognitive map decision support system for successful triage to reduce unnecessary emergency room admissions for the elderly In Fuzziness and Medicine: Philosophical Reflections and Application Systems in Health Care 2013 (pp. 415–436). Springer, Berlin, Heidelberg.

[16] Wang ST. Construct an optimal triage prediction model: A case study of the emergency department of a teaching hospital in Taiwan. Journal of medical systems. 2013 10 1; 37(5):9968 10.1007/s10916-013-9968-x 23990379

[17] Lin WT, Wang ST, Chiang TC, Shi YX, Chen WY, Chen HM. Abnormal diagnosis of Emergency Department triage explored with data mining technology: An Emergency Department at a Medical Center in Taiwan taken as an example. Expert Systems with Applications. 2010 4 1;37(4):2733–41. 10.1016/j.eswa.2009.08.006

[18] Fields EB, Okudan GE, Ashour OM. Rank aggregation methods comparison: A case for triage prioritization. Expert Systems with Applications. 2013 3 1; 40(4):1305–11. 10.1016/j.eswa.2012.08.060

[19] Georgopoulos VC, Stylios CD. Supervisory fuzzy cognitive map structure for triage assessment and decision support in the emergency department In Simulation and Modeling Methodologies, Technologies and Applications 2015 (pp. 255–269). Springer, Cham.

[20] Georgopoulos VC, Stylios CD. Fuzzy cognitive maps for decision making in triage of non-critical elderly patients. In 2017 International Conference on Intelligent Informatics and Biomedical Sciences (ICIIBMS) 2017 Nov 24 (pp. 225-228). IEEE.

[21] Georgopoulos VC, Stylios CD. Introducing fuzzy cognitive maps for developing decision support system for triage at emergency room admissions for the elderly. IFAC Proceedings Volumes. 2012 1 1; 45(18):484–9. 10.3182/20120829-3-HU-2029.00107

[22] Soufi MD, Samad-Soltani T, Vahdati SS, Rezaei-Hachesu P. Decision support system for triage management: A hybrid approach using rule-based reasoning and fuzzy logic. International journal of medical informatics. 2018 6 1; 114:35–44. 10.1016/j.ijmedinf.2018.03.008 29673601

[23] Lin WT, Jou YT, Wu YC, Hsiao YD. Data Mining Applied to the Predictive Model of Triage System in Emergency Department. In Proceedings of World Academy of Science, Engineering and Technology 2013 Jan 1 (No. 78, p. 1789). World Academy of Science, Engineering and Technology (WASET).

[24] Teubner DJ, Considine J, Hakendorf P, Kim S, Bersten AD. Model to predict inpatient mortality from information gathered at presentation to an emergency department: The Triage Information Mortality Model (TIMM). Emergency Medicine Australasia. 2015; 27(4):300–306. 10.1111/1742-6723.12425 26147765

[25] Coslovsky M, Takala J, Exadaktylos AK, Martinolli L, Merz TM. A clinical prediction model to identify patients at high risk of death in the emergency department. Intensive care medicine. 2015; 41(6):1029–1036. 10.1007/s00134-015-3737-x 25792208 PMC4477719

[26] Plunkett PK, Byrne DG, Breslin T, Bennett K, Silke B. Increasing wait times predict increasing mortality for emergency medical admissions. European Journal of Emergency Medicine. 2011 8 1; 18(4):192–6. 10.1097/MEJ.0b013e328344917e 21317786

[27] Raita Y, Goto T, Faridi MK, Brown DF, Camargo CA, Hasegawa K. Emergency department triage prediction of clinical outcomes using machine learning models. Critical Care. 2019; 23(1):64 10.1186/s13054-019-2351-7 30795786 PMC6387562

[28] LaMantia MA, Stewart PW, Platts-Mills TF, Biese KJ, Forbach C, Zamora E, et al Predictive value of initial triage vital signs for critically ill older adults. Western Journal of Emergency Medicine. 2013; 14(5):453 10.5811/westjem.2013.5.13411 24106542 PMC3789908

[29] Barfod C, et al Abnormal vital signs are strong predictors for intensive care unit admission and in-hospital mortality in adults triaged in the emergency department-a prospective cohort study. Scandinavian journal of trauma, resuscitation and emergency medicine. 2012; 20(1):28 10.1186/1757-7241-20-28 22490208 PMC3384463

[30] Barnes S, Saria S, Levin S. An evolutionary computation approach for optimizing multilevel data to predict patient outcomes. Journal of healthcare engineering. 2018;2018 10.1155/2018/7174803 29744026 PMC5878885

[31] Jenny MA, et al Are mortality and acute morbidity in patients presenting with nonspecific complaints predictable using routine variables? Academic Emergency Medicine. 2015 10; 22(10):1155–63. 10.1111/acem.12755 26375290

[32] Lee DS, Stitt A, Austin PC, Stukel TA, Schull MJ, Chong A, et al Prediction of heart failure mortality in emergent care: a cohort study. Annals of internal medicine. 2012 6 5; 156(11):767–75. 10.7326/0003-4819-156-11-201206050-00003 22665814

[33] Ong ME, et al Prediction of cardiac arrest in critically ill patients presenting to the emergency department using a machine learning score incorporating heart rate variability compared with the modified early warning score. Critical Care. 2012 6 1; 16(3):R108 10.1186/cc11396 22715923 PMC3580666

[34] Hong W, Earnest A, Sultana P, Koh Z, Shahidah N, Ong ME. How accurate are vital signs in predicting clinical outcomes in critically ill emergency department patients. European Journal of Emergency Medicine. 2013 2 1; 20(1):27–32. 10.1097/MEJ.0b013e32834fdcf3 22198158

[35] Ljunggren M, Castrén M, Nordberg M, Kurland L. The association between vital signs and mortality in a retrospective cohort study of an unselected emergency department population. Scandinavian journal of trauma, resuscitation and emergency medicine. 2016 12; 24(1):21 10.1186/s13049-016-0213-8 26940235 PMC4778316

[36] Djärv T, Castrén M, Mårtenson L, Kurland L. Decreased general condition in the emergency department: high in-hospital mortality and a broad range of discharge diagnoses. European Journal of Emergency Medicine. 2015 8 1; 22(4):241–6. 10.1097/MEJ.0000000000000164 24910961

[37] Ro YS, Shin SD, Song KJ, Cha WC, Cho JS. Triage-based resource allocation and clinical treatment protocol on outcome and length of stay in the emergency department. Emergency Medicine Australasia. 2015 8; 27(4):328–35. 10.1111/1742-6723.12426 26075591

[38] LaMantia MA, et al Predicting hospital admission and returns to the emergency department for elderly patients. Academic emergency medicine. 2010 3; 17(3):252–9. 10.1111/j.1553-2712.2009.00675.x 20370757 PMC5985811

[39] Zlotnik A, Alfaro MC, Pérez MC, Gallardo-Antolín A, Martínez JM. Building a decision support system for inpatient admission prediction with the Manchester triage system and administrative check-in variables. CIN: Computers, Informatics, Nursing. 2016 5 1; 34(5):224–30. 10.1097/CIN.0000000000000230 26974710

[40] Cameron A, Rodgers K, Ireland A, Jamdar R, McKay GA. A simple tool to predict admission at the time of triage. Emergency Medicine Journal. 2015 3 1; 32(3):174–9. 10.1136/emermed-2013-203200 24421344 PMC4345772

[41] Graham B, Bond R, Quinn M, Mulvenna M. Using data mining to predict hospital admissions from the emergency department. IEEE Access. 2018; 6:10458–10469. 10.1109/ACCESS.2018.2808843

[42] Zhang X, Kim J, Patzer RE, Pitts SR, Patzer A, Schrager JD. Prediction of emergency department hospital admission based on natural language processing and neural networks. Methods of information in medicine. 2017; 56(05):377–89. 10.3414/ME17-01-0024 28816338

[43] Handly N, Thompson DA, Li J, Chuirazzi DM, Venkat A. Evaluation of a hospital admission prediction model adding coded chief complaint data using neural network methodology. European Journal of Emergency Medicine. 2015 4 1;22(2):87–91. 10.1097/MEJ.0000000000000126 24509606

[44] Ruger JP, Lewis LM, Richter CJ. Identifying high-risk patients for triage and resource allocation in the ED. The American journal of emergency medicine. 2007 9 1; 25(7):794–8. 10.1016/j.ajem.2007.01.014 17870484

[45] Hendin A, Eagles D, Myers V, Stiell IG. Characteristics and outcomes of older emergency department patients assigned a low acuity triage score. Canadian Journal of Emergency Medicine. 2018 9; 20(5):762–9. 10.1017/cem.2018.17 29502553

[46] Sun Y, Heng BH, Tay SY, Seow E. Predicting hospital admissions at emergency department triage using routine administrative data. Academic Emergency Medicine. 2011 8; 18(8):844–50. 10.1111/j.1553-2712.2011.01125.x 21843220

[47] Araz OM, Olson D, Ramirez-Nafarrate A. Predictive analytics for hospital admissions from the emergency department using triage information. International Journal of Production Economics. 2019 2 1; 208:199–207. 10.1016/j.ijpe.2018.11.024

[48] Hong WS, Haimovich AD, Taylor RA. Predicting hospital admission at emergency department triage using machine learning. PloS one. 2018; 13(7). 10.1371/journal.pone.0201016 PMC6054406 30028888

[49] Parker CA, Liu N, Wu SX, Shen Y, Lam SSW, Ong MEH. Predicting hospital admission at the emergency department triage: A novel prediction model. The American journal of emergency medicine (2018). 2018. 10.1016/j.ajem.2018.10.060 30413365

[50] Ng CJ, Liao PJ, Chang YC, Kuan JT, Chen JC, Hsu KH. Predictive factors for hospitalization of nonurgent patients in the emergency department. Medicine. 2016 6; 95(26). 10.1097/MD.0000000000004053 PMC4937954 27368040

[51] Kim SW, Li JY, Hakendorf P, Teubner DJ, Ben-Tovim DI, Thompson CH. Predicting admission of patients by their presentation to the emergency department. Emergency Medicine Australasia. 2014 8; 26(4):361–7. 10.1111/1742-6723.12252 24934833

[52] Handly N, Thompson DA, Venkat A. Derivation and validation of a hospital admission prediction model adding coded chief complaint to demographic, emergency department operational and patient acuity data available at emergency department triage using neural net methodology. Annals of Emergency Medicine. 2013 10 1; 62(4):S138 10.1016/j.annemergmed.2013.07.212

[53] Srivilaithon W, Amnuaypattanapon K, Limjindaporn C, Imsuwan I, Daorattanachai K, Dasanadeba I, et al Predictors of in-hospital cardiac arrest within 24 h after emergency department triage: A case–control study in urban Thailand. Emergency Medicine Australasia. 2019 10; 31(5):843–50. 10.1111/1742-6723.13267 30887710

[54] Pereira L, et al Unscheduled-return-visits after an emergency department (ED) attendance and clinical link between both visits in patients aged 75 years and over: a prospective observational study. PloS one. 2015; 10(4). 10.1371/journal.pone.0123803 PMC4390330 25853822

[55] Gligorijevic D, et al. Deep attention model for triage of emergency department patients. In Proceedings of the 2018 SIAM International Conference on Data Mining 2018 May 7 (pp. 297-305). Society for Industrial and Applied Mathematics.

[56] Fernandes M, Vieira SM, Leite F, Palos C, Finkelstein S, Sousa JM. Clinical Decision Support Systems for Triage in the Emergency Department using Intelligent Systems: a Review. Artificial Intelligence in Medicine. 2019 11 17:101762 10.1016/j.artmed.2019.101762 31980099

[57] Berchet C, et al Emergency Care Services: Trends, Drivers and Interventions to Manage the Demand. OECD Publishing; 2015.

[58] Cosgriff, Christopher V., et al. Developing well-calibrated illness severity scores for decision support in the critically ill. npj Digital Medicine 2.1 (2019): 1-8. 10.1038/s41746-019-0153-6 PMC6695410 31428687

[59] Pappachan, John V., et al. Comparison of outcome from intensive care admission after adjustment for case mix by the APACHE III prognostic system. Chest 115.3 (1999): 802-810. 10.1378/chest.115.3.802 10084495

[60] Rowan Kathryn M., et al Intensive Care Society’s Acute Physiology and Chronic Health Evaluation (APACHE II) study in Britain and Ireland: a prospective, multicenter, cohort study comparing two methods for predicting outcome for adult intensive care patients. Critical care medicine 22 9 (1994): 1392–1401. 10.1097/00003246-199409000-00007 8062560

[61] Rowan K. M., et al Intensive Care Society’s APACHE II study in Britain and Ireland–II: Outcome comparisons of intensive care units after adjustment for case mix by the American APACHE II method. Bmj 307 6910 (1993): 977–981. 8241909 10.1136/bmj.307.6910.977 PMC1679167

[62] Beam Andrew L., and Kohane Isaac S. Big data and machine learning in health care. Jama 319 13 (2018): 1317–1318. 10.1001/jama.2017.18391 29532063

[63] Breiman L. Random forests. Machine learning. 2001; 45(1):5–32. 10.1023/A:1010933404324

[64] Friedman Jerome H. Greedy function approximation: a gradient boosting machine Annals of statistics. JSTOR, 2001;1189–1232.

[65] Chen, Tianqi, and Carlos Guestrin. Xgboost: A scalable tree boosting system. Proceedings of the 22nd acm sigkdd international conference on knowledge discovery and data mining. ACM, 2016.

[66] Azari A, Janeja VP, Levin S. Imbalanced learning to predict long stay Emergency Department patients. In: Bioinformatics and Biomedicine (BIBM), 2015 IEEE International Conference on. IEEE; 2015. p. 807–814.

[67] Cohen J. A coefficient of agreement for nominal scales. Educational and psychological measurement. 1960; 20(1):37–46. 10.1177/001316446002000104

[68] McHugh ML. Interrater reliability: the kappa statistic. Biochemia medica: Biochemia medica. 2012; 22(3):276–282. 10.11613/BM.2012.031 23092060 PMC3900052

[69] Brownlee J. Imbalanced Classification with Python: Better Metrics, Balance Skewed Classes, Cost-Sensitive Learning. Machine Learning Mastery 2020. Accessed 5/2/2020. url=https://books.google.pt/books?id=jaXJDwAAQBAJ.

