# Supplementary material for: Risk of mortality and cardiopulmonary arrest in critical patients presenting to the emergency department using machine learning and natural language processing
Source: PLoS One. 2020 Apr 2;15(4):e0230876. doi: 10.1371/journal.pone.0230876 (PMC7117713; doi:10.1371/journal.pone.0230876)
Supplement: S1 Appendix — (PDF) [file pone.0230876.s001.pdf]

Detailed inclusion and exclusion criteria

Duplicated triages – triage information registered more than once in the same episode, with exactly the same parameters values, may happen for example when there is a change in triage priority, or additional information is registered for the patient. Duplicated triages in a 24 hour period were excluded, since training and testing the model with duplicated information from patients could introduce model bias. Clinical protocols may be activated at the triage stage, such as sepsis, stroke, trauma, thoracic pain, anti-hypertensive, analgesia, antipyretic and critical patient protocol specific from HBA. Triages with activation of protocols were excluded from the study, since the intervention for this cohort of patients is immediate and therefore they are not in the same conditions as the other patients in the study. Obstetric patients, patients with data inconsistencies such as unknown age, unknown priority, unknown time of admission in the ED and referrals to other hospital ED in the 24 hour period were excluded.
