# Supplementary material for: Risk of mortality and cardiopulmonary arrest in critical patients presenting to the emergency department using machine learning and natural language processing
Source: PLoS One. 2020 Apr 2;15(4):e0230876. doi: 10.1371/journal.pone.0230876 (PMC7117713; doi:10.1371/journal.pone.0230876)
Supplement: S2 Appendix — (PDF) [file pone.0230876.s002.pdf]

Data preprocessing

The data consisted of clinical and demographic variables collected at triage in the emergency department. For continuous variables min-max scaler normalization was applied. The criteria for identification of outliers and abnormal values in each physiological parameter was based on values not within the medical physiological ranges [1–3]. Only values not possible to exist in a patient were excluded. For each physiological variable, a dummy indicator variable was created and 1 was imputed each time an abnormal value was identified in the correspondent variable for the patient. In the same way, for each physiological variable, a dummy indicator variable was created and 1 was imputed each time there was a missing value in the correspondent variable for the patient. The last present value was imputed with data collected in a time frame [-60, 15] min, with triage as reference (0 min), since the values can be registered at a different time due to a malfunction in the registration system. The mean value of the population was imputed for the remaining missing data. The chief complaint consisted of unstructured free text information and it was subjected to lowercasing, a process of temporal normalization, tokenization, expansion of abbreviations and correction using Jaro-Winkler and stemming. The corpus of tokens was created recurring to Firefox dictionary for language tokens as well as sources for sublanguage [4] and for medications [5].

References

1. Plus Medline;. https://medlineplus.gov/vitalsigns.html.
2. Medscape; https://emedicine.medscape.com/article/2172054-overview.
3. Salmasi V, Maheshwari K, Yang D, Mascha EJ, Singh A, Sessler DI, et al. Relationship between Intraoperative Hypotension, Defined by Either Reduction from Baseline or Absolute Thresholds, and Acute Kidney and Myocardial Injury after Noncardiac Surgery A Retrospective Cohort Analysis. Anesthesiology: The Journal of the American Society of Anesthesiologists. 2017;126(1):47–65.
4. Association of Medical Doctors of Portugal, https://www.medicosdeportugal.pt/glossario/.
5. Infarmed National Authority of Medications and Health Products,http://www.infarmed.pt/.
