# Supplementary material for: Risk of mortality and cardiopulmonary arrest in critical patients presenting to the emergency department using machine learning and natural language processing
Source: PLoS One. 2020 Apr 2;15(4):e0230876. doi: 10.1371/journal.pone.0230876 (PMC7117713; doi:10.1371/journal.pone.0230876)
Supplement: S1 Table — DBP—diastolic blood pressure. (1) values not within normal range. (PDF) [file pone.0230876.s003.pdf]

Table S1. Criteria to exclude outliers and identify abnormal values in patients' variables.

Variables	Normal range	Exclusion criteria	Identification criteria	Unit
Glasgow coma scale	3-15	<3, >15	-	-
Pain scale	0-10	<0, >10	-	-
Respiratory rate	16-20	> 80	(1)	breaths/min
Heart rate	60-100	< 0, >300	(1)	beats/min
Temperature	35-38	<20, > 45	<35, >38.5	°C
Oximetry	95-100	<50, >100	<95	%
Systolic blood pressure	90-140	≥ 300, ≤ 20, $\leq$ DBP+5	(1)	mmHg
Diastolic blood pressure	60-90	≤ 5, ≥ 200	(1)	mmHg
Glycemia	70-99	<0	<55, >200	mg/dL

DBP - diastolic blood pressure. (1) values not within normal range.
