# Supplementary material for: Risk of mortality and cardiopulmonary arrest in critical patients presenting to the emergency department using machine learning and natural language processing
Source: PLoS One. 2020 Apr 2;15(4):e0230876. doi: 10.1371/journal.pone.0230876 (PMC7117713; doi:10.1371/journal.pone.0230876)
Supplement: S2 Table — “Warm start” (reusing the solution of the previous call to fit as initialization) was varied between True/False for the three modeling techniques. (PDF) [file pone.0230876.s004.pdf]

Table S2. Hyperparameter optimization in random search 10-fold cross validation. "Warm start" (reusing the solution of the previous call to fit as initialization) was varied between True/False for the three modeling techniques.

**Technique**	**Hyperparameters**
Logistic regression	The LR regularization constant was varied for the values 0.0001, 0.001, 0.01, 0.1, 1.0.<br>The limited-memory solver was varied between "newton-cg", "lbfgs", "sag" and "saga" with L2 penalization.
Random forests	Number of trees was varied for the values {100, 150, 200, 250, 300, 350}.<br>The maximum tree depth was varied for the values {2, 3, 4, 5}.<br>The creation of trees was varied between using bootstrap samples or the whole data to fit the model.
Extreme gradient boosting	Number of trees was varied for the values {100, 150, 200, 250, 300, 350}.<br>Learning rate was varied for the values {0.01, 0.05, 0.06, 0.07, 0.08, 0.09, 0.1}.<br>Gamma specifies the minimum loss reduction required to make a split and it was varied for the values {0, 1, 5}.<br>The maximum tree depth was varied for the values {2, 3, 4, 5}.<br>Percentage of features selected to build a tree was varied for the values {30, 40, 50, 60, 70, 80}.<br>Percentage of patients in train selected to build a tree was varied for the values {80, 90, 100}.
TF-IDF	The number of words in a patient's chief complaint was varied between unigrams (a single word), bigrams (two consecutive words) and trigrams (three consecutive words), as following: unigrams, bigrams, triagrams, unigrams + bigrams, bigrams + trigrams and unigrams + bigrams + trigrams.<br>The maximum number of words to consider from the vocabulary was varied for the values {1000, 9500, 15000, 25000, 29000} as well as considering all the vocabulary in train.
