# Supplementary material for: Risk of mortality and cardiopulmonary arrest in critical patients presenting to the emergency department using machine learning and natural language processing
Source: PLoS One. 2020 Apr 2;15(4):e0230876. doi: 10.1371/journal.pone.0230876 (PMC7117713; doi:10.1371/journal.pone.0230876)
Supplement: S3 Table — (PDF) [file pone.0230876.s005.pdf]

Table S3. Number of missing and abnormal vitals, Glasgow coma Scale, first visit indicator and types of exams used for modeling summarized for emergency department patients with and without the composite outcome.

	**Composite outcome**	
	**Yes (N=1121)**	**No (N=234711)**
Missing vitals		
0	345 (31)	29462 (13)
1	97 (9)	22364 (10)
2	60 (5)	14896 (6)
3	101 (9)	36440 (16)
4	70 (6)	20496 (9)
5	142 (13)	71835 (31)
6	306 (27)	39218 (17)
Abnormal vitals		
0	402 (36)	137662 (58.7)
1	236 (21)	50153 (21.4)
2	188 (17)	29786 (12.7)
3	175 (16)	12718 (5.4)
4	85 (8)	3831 (1.6)
5	35 (3)	561 (0.2)
Glasgow Coma Scale		
3	75 (7)	48 (0.02)
4	9 (1)	20 (0.01)
5	7 (1)	33 (0.01)
6	25 (2)	124 (0.05)
7	25 (2)	210 (0.09)
8	40 (3)	374 (0.16)
9	65 (6)	740 (0.32)
10	63 (6)	1293 (0.55)
11	43 (4)	1518 (0.65)
12	38 (3)	1288 (0.55)
13	37 (3)	1426 (0.61)
14	67 (6)	7380 (3.14)
15	627 (56)	220257 (93.84)
Ophthalmology exam		
No	1121 (100)	232464 (99)
Yes	0 (0)	2247 (1)
Otolaryngology exam		
No	1121 (100)	234221 (99.8)
Yes	0 (0)	490 (0.2)
Electrocardiogram		
No	1111 (99.1)	230654(98.3)
Yes	10 (0.89)	4057(1.7)
X-ray		
No	1121 (100)	234607 (99.96)
Yes	0 (0)	104 (0.04)
Orthopedic exam		
No	1119 (99.8)	219961 (93.7)
Yes	2 (0.2)	14750(6.3)
First ED visit		
No	784 (70)	120557 (51)
Yes	337 (30)	114154 (49)

The table shows number of patients and the figures in parentheses are the column percentages within each categorical variable.
