# Supplementary material for: Risk of mortality and cardiopulmonary arrest in critical patients presenting to the emergency department using machine learning and natural language processing
Source: PLoS One. 2020 Apr 2;15(4):e0230876. doi: 10.1371/journal.pone.0230876 (PMC7117713; doi:10.1371/journal.pone.0230876)
Supplement: S4 Table — (PDF) [file pone.0230876.s006.pdf]

Table S4. Time variables used for modeling summarized for emergency department patients with and without the composite outcome.

	Composite outcome	
	**Yes (N=1121)**	**No (N=234711)**
Hour		
12am	31 (3)	6308 (3)
1am	22 (2)	5385 (2)
2am	14 (1)	4151 (2)
3am	23 (2)	3451 (1)
4am	13 (1)	3179 (1)
5am	17 (2)	2958 (1)
6am	14 (1)	3314 (1)
7am	21 (2)	4274 (2)
8am	29 (3)	7070 (3)
9am	30 (3)	11556 (5)
10am	79 (7)	15365 (7)
11am	95 (8)	16602 (7)
12am	85 (8)	14233 (6)
13am	74 (7)	13098 (6)
14am	79 (7)	14664 (6)
15am	56 (5)	15445 (7)
16am	66 (6)	14234 (6)
17am	64 (6)	12634 (5)
18am	64(6)	12281(5)
19am	65 (6)	12592 (5)
20am	48 (4)	11463 (5)
21am	42 (4)	11792 (5)
22am	49(4)	10489(4)
23am	41 (4)	8173 (3)
Month		
January	129 (12)	19283 (8)
February	103 (9)	17604 (8)
March	123 (11)	21711 (9)
April	74(7)	20269 (9)
May	86 (8)	22375 (10)
June	93 (8)	21849 (9)
July	118 (11)	22288 (9)
August	57 (5)	17035 (7)
September	69 (6)	17578 (7)
October	80 (7)	18380 (8)
November	74 (7)	17843 (8)
December	115 (10)	18496 (8)
Weekday		
Monday	161 (14)	38041 (16)
Tuesday	168(15)	35227 (15)
Wednesday	152 (14)	34420 (15)
Thursday	156 (14)	34079 (15)
Friday	161 (14)	33702 (14)
Saturday	142 (13)	29852 (13)
Sunday	181 (16)	29390 (13)

The table shows number of patients and the figures in parentheses are the column percentages within each categorical variable.
