# Supplementary material for: Risk of mortality and cardiopulmonary arrest in critical patients presenting to the emergency department using machine learning and natural language processing
Source: PLoS One. 2020 Apr 2;15(4):e0230876. doi: 10.1371/journal.pone.0230876 (PMC7117713; doi:10.1371/journal.pone.0230876)
Supplement: S5 Table — (PDF) [file pone.0230876.s007.pdf]

Table S5. Abnormal and missing values for vital signs used for modeling summarized for emergency department patients with and without the composite outcome.

	Composite outcome	
	**Yes (N=1121)**	**No (N=234711)**
Pain scale missing		
1 (yes)	552 (49)	25521 (11)
0 (no)	569 (51)	209190 (89)
Heart rate missing		
1 (yes)	445 (40)	114620 (49)
0 (no)	676 (60)	120091 (51)
Diastolic blood pressure missing		
1 (yes)	643 (57)	167789 (71)
0 (no)	478 (43)	66922 (29)
Systolic blood pressure missing		
1 (yes)	641 (57)	167487 (71)
0 (no)	480 (43)	67224 (29)
Temperature missing		
1 (yes)	447 (40)	50495 (22)
0 (no)	674 (60)	184216 (78)
Glycemia missing		
1 (yes)	739 (66)	168675 (72)
0 (no)	382 (34)	66036 (28)
Glasgow Coma Scale missing		
1 (yes)	376 (33.5)	55221 (24)
0 (no)	745 (66.5)	179490 (76)
Pulse oximetry missing		
1 (yes)	493 (44)	145769 (62)
0 (no)	628 (56)	88942 (38)
Respiratory rate missing		
1 (yes)	677 (60)	191783 (82)
0 (no)	444 (40)	42928 (18)
Abnormal heart rate		
1 (yes)	456 (41)	66345 (28)
0 (no)	665 (59)	168366 (72)
Abnormal diastolic blood pressure		
1 (yes)	231 (21)	19951 (9)
0 (no)	890 (79)	214760 (91)
Abnormal systolic blood pressure		
1 (yes)	188 (17)	32026 (14)
0 (no)	933 (83)	202685 (86)
Abnormal temperature		
1 (yes)	93 (8)	6814 (3)
0 (no)	1028 (92)	227897 (97)
Abnormal glycemia		
1 (yes)	137 (12)	9387 (4)
0 (no)	984 (88)	225324 (96)
Abnormal pulse oximetry		
1 (yes)	492 (44)	22618 (10)
0 (no)	629 (56)	212093 (90)
Abnormal respiratory rate		
1 (yes)	285 (25)	25068 (11)
0 (no)	836 (75)	209643 (89)

The table shows number of patients and the figures in parentheses are the column percentages within each categorical variable.
