# Supplementary material for: Risk of mortality and cardiopulmonary arrest in critical patients presenting to the emergency department using machine learning and natural language processing
Source: PLoS One. 2020 Apr 2;15(4):e0230876. doi: 10.1371/journal.pone.0230876 (PMC7117713; doi:10.1371/journal.pone.0230876)
Supplement: S6 Table — (PDF) [file pone.0230876.s008.pdf]

Table S6. Pain scale used for modeling summarized for emergency department patients with and without the composite outcome.

	Composite outcome	
	**Yes (N=1121)**	**No (N=234711)**
Pain scale		
0	348 (31.0)	43828 (18.7)
1	13 (1.2)	2522 (1.1)
2	21 (1.9)	8347 (3.5)
3	19 (1.7)	13759 (5.9)
4	572 (51.0)	40393 (17.2)
5	71 (6.3)	90123 (38.4)
6	23 (2.0)	19677 (8.4)
7	9 (0.8)	3788 (1.6)
8	40 (3.6)	11273 (4.8)
9	4 (0.4)	848 (0.3)
10	1 (0.1)	153 (0.1)

The table shows number of patients and the figures in parentheses are the column percentages within each categorical variable.
