# Supplementary material for: Risk of mortality and cardiopulmonary arrest in critical patients presenting to the emergency department using machine learning and natural language processing
Source: PLoS One. 2020 Apr 2;15(4):e0230876. doi: 10.1371/journal.pone.0230876 (PMC7117713; doi:10.1371/journal.pone.0230876)
Supplement: S7 Table — In brackets is the result for 100 bootstrapping iterations in 95% confidence intervals. (PDF) [file pone.0230876.s009.pdf]

Table S7. Average modeling performance results in test. In brackets is the result for 100 bootstrapping iterations in 95% confidence intervals.

Model	AUROC	AUPRC	AP	Specificity	Recall	Precision	F1-score	κ
**Reference model**								
LR	0.85 [0.82-0.87]	0.28 [0.24-0.32]	0.10 [0.07-0.12]	0.82 [0.82-0.82]	0.82 [0.77-0.85]	0.02 [0.02-0.02]	0.04 [0.04-0.05]	0.03 [0.03-0.04]
**All predictors except chief complaint**								
LR	0.93 [0.92-0.94]	0.17 [0.15-0.21]	0.17 [0.15-0.21]	0.91 [0.91-0.91]	0.80 [0.75-0.84]	0.04 [0.04-0.05]	0.08 [0.07-0.09]	0.07 [0.06-0.08]
RF	0.95 [0.93-0.96]	0.21 [0.17-0.25]	0.21 [0.17-0.25]	0.88 [0.87-0.88]	0.86 [0.82-0.89]	0.03 [0.03-0.04]	0.06 [0.06-0.07]	0.05 [0.05-0.06]
XGBoost	0.96 [0.95-0.97]	0.25 [0.21-0.31]	0.26 [0.22-0.31]	0.93 [0.93-0.93]	0.84 [0.80-0.88]	0.05 [0.05-0.06]	0.10 [0.09-0.11]	0.09 [0.08-0.10]
**All predictors including chief complaint**								
LR	0.95 [0.94-0.96]	0.25 [0.21-0.30]	0.25 [0.21-0.30]	0.88 [0.88-0.88]	0.86 [0.83-0.90]	0.03 [0.03-0.04]	0.06 [0.06-0.07]	0.06 [0.05-0.06]
RF	0.94 [0.93-0.95]	0.20 [0.16-0.24]	0.21 [0.16-0.24]	0.89 [0.89-0.89]	0.85 [0.81-0.88]	0.04 [0.03-0.04]	0.07 [0.06-0.08]	0.06 [0.05-0.07]
XGBoost	0.96 [0.95-0.97]	0.30 [0.25-0.35]	0.30 [0.25-0.35]	0.94 [0.93-0.94]	0.84 [0.80-0.88]	0.06 [0.05-0.07]	0.11 [0.10-0.12]	0.10 [0.09-0.11]

Abbreviations: LR - Logistic regression, RF - Random forests, XGBoost - extreme gradient boosting, AUROC - area under the ROC curve, AUPRC - area under the precision recall curve, AP - Average precision.
