# Supplementary figures and images for: Risk of mortality and cardiopulmonary arrest in critical patients presenting to the emergency department using machine learning and natural language processing
Source: PLoS One. 2020 Apr 2;15(4):e0230876. doi: 10.1371/journal.pone.0230876 (PMC7117713; doi:10.1371/journal.pone.0230876)

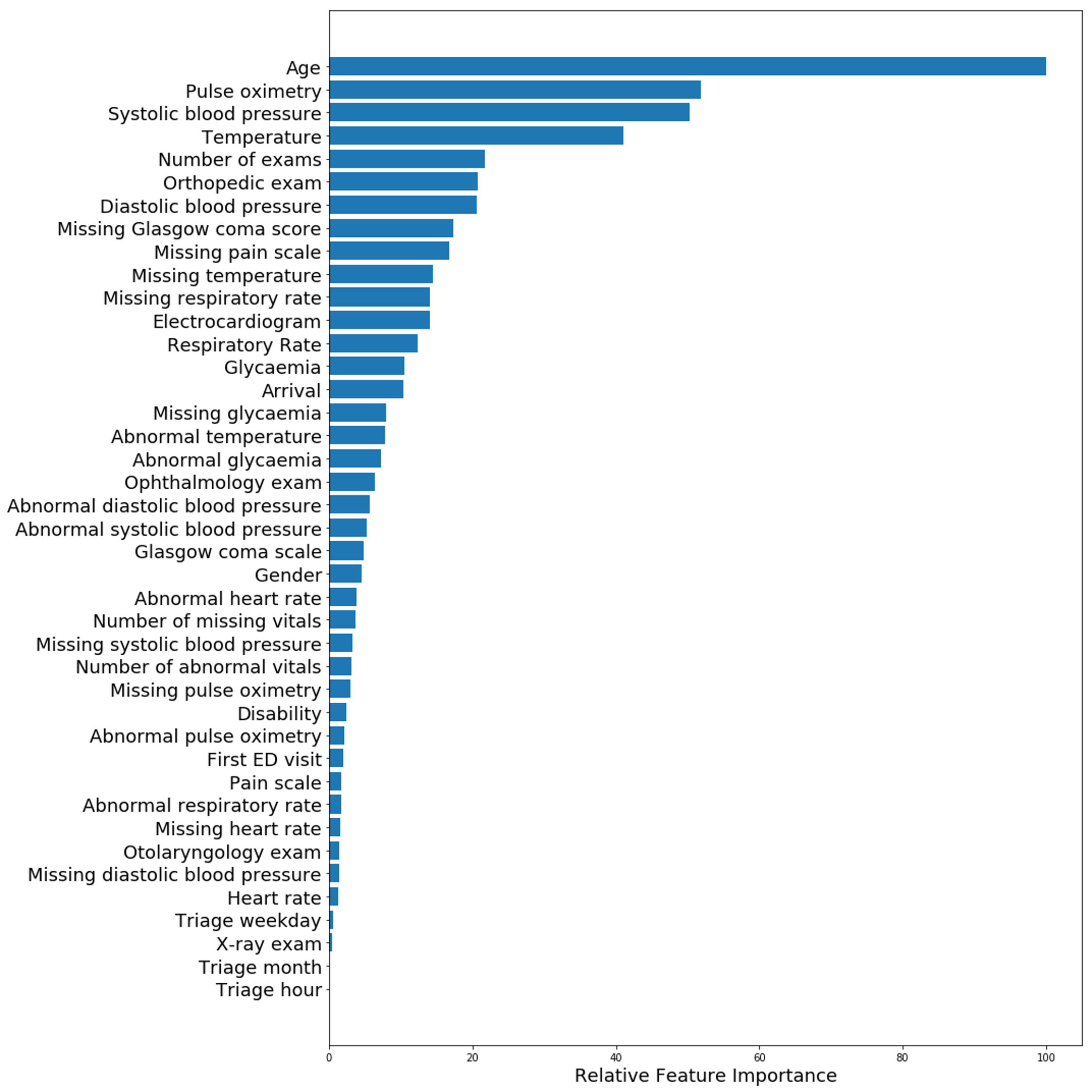

Supplement: S1 Fig — (TIF) [file pone.0230876.s010.tif]

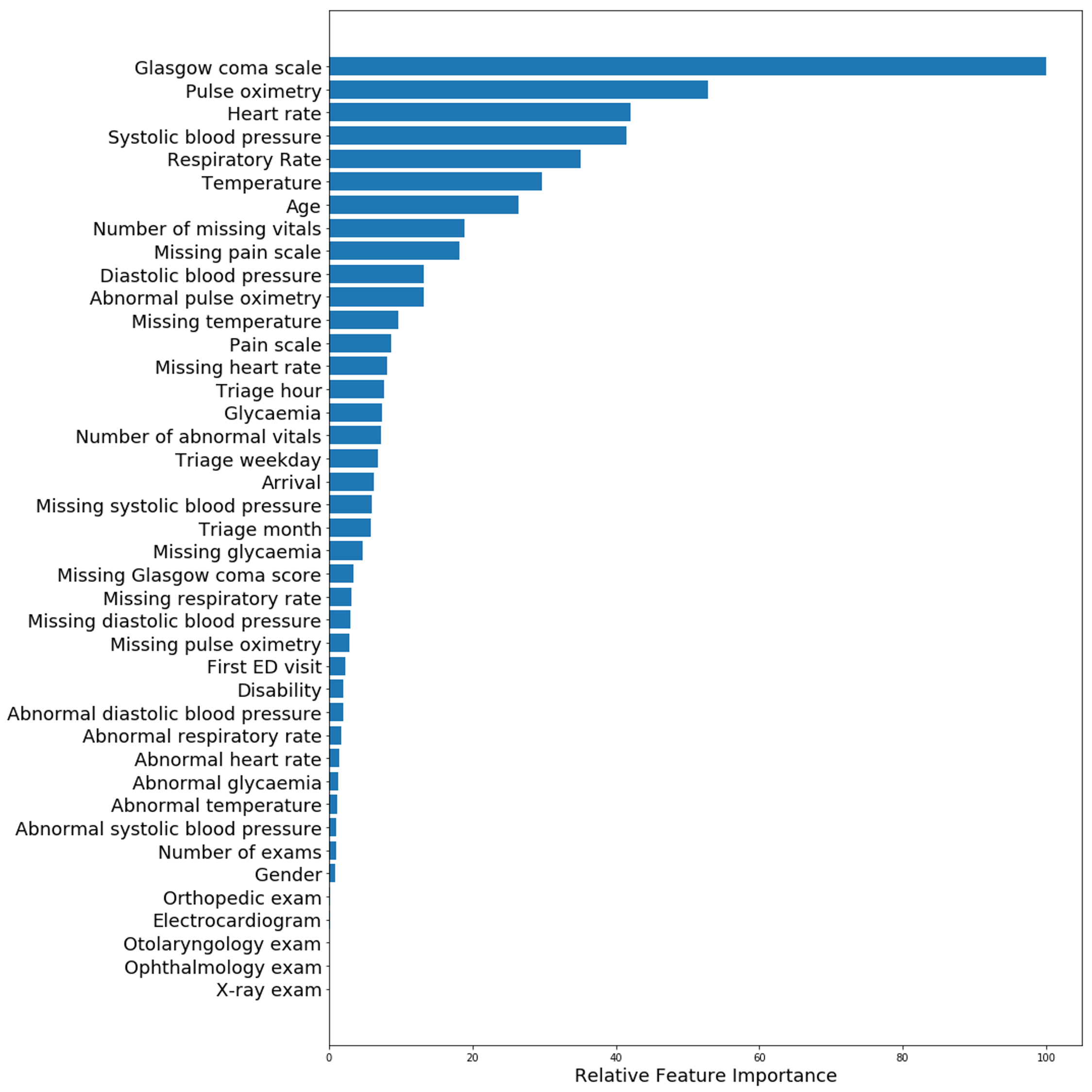

Supplement: S2 Fig — (TIF) [file pone.0230876.s011.tif]

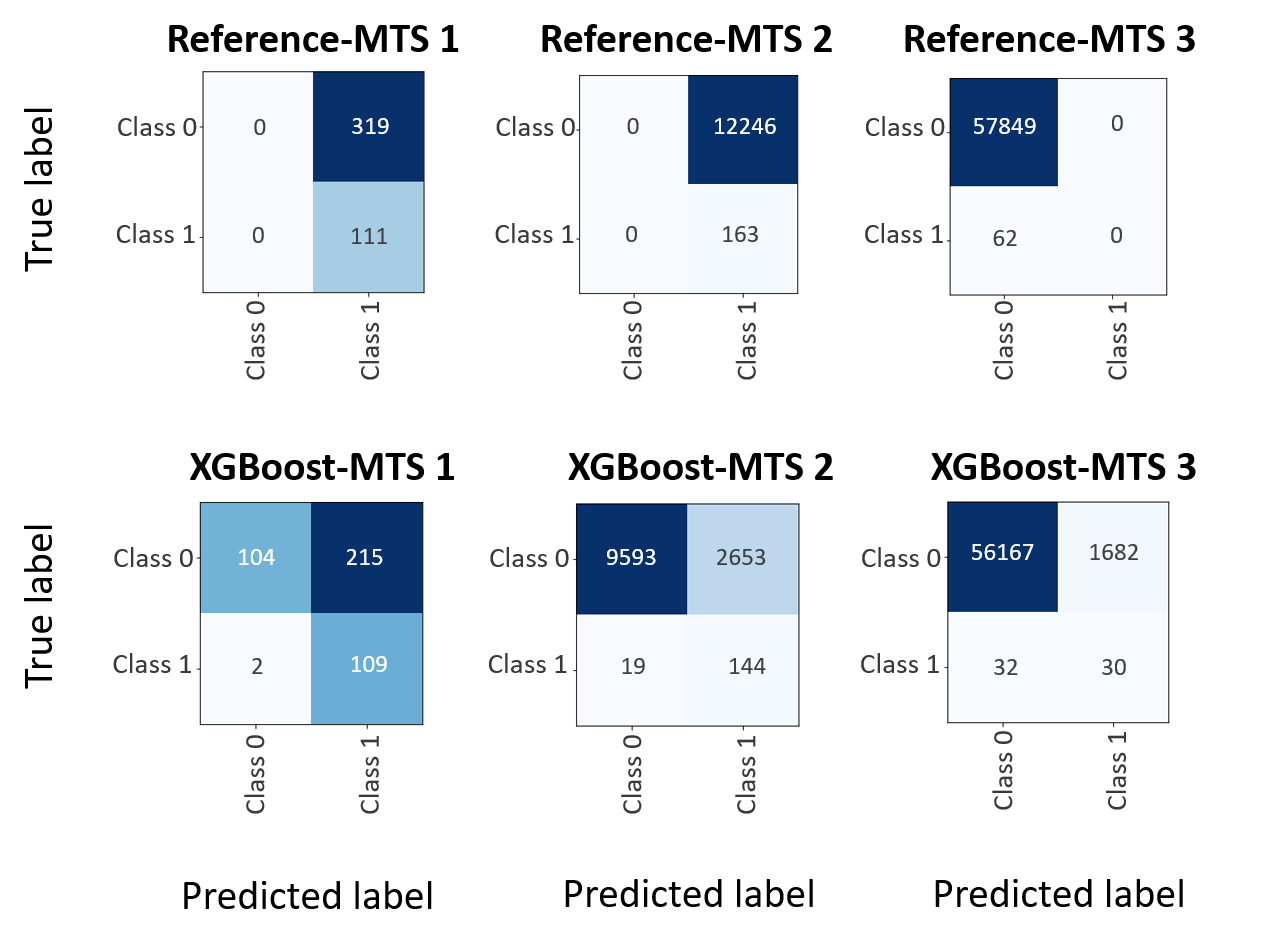

Supplement: S3 Fig — (TIF) [file pone.0230876.s012.tif]
